# Influence of geography, seasonality and experimental selection on *Chironomus riparius* recombination rates

**DOI:** 10.1186/s12864-026-12809-5

**Published:** 2026-04-15

**Authors:** María Esther Nieto-Blázquez, Cosima Caliendo, Laura C. Pettrich, Ann-Marie Waldvogel, Markus Pfenninger

**Affiliations:** 1 Department of Molecular Ecology, Senckenberg Biodiversity and Climate Research Centre, Frankfurt am Main, Germany; 2 Institute of Human Genetics, University Medical Center, Johannes Gutenberg University Mainz, Staudinger Weg, Mainz, Germany; 3 Institute of Zoology, University of Cologne, Cologne, Germany; 4 School of Life Sciences, Technical University of Munich, Limnological Research Station, Hofmark, Iffeldorf, Germany; 5 Institute for Molecular and Organismic Evolution, Johannes Gutenberg University, Johann-Joachim-Becher-Weg, Mainz, Germany

**Keywords:** Recombination rates, Geography, Seasonality, Experimental selection, Genetic diversity, GC content, F_ST_, Transposable elements, Structural equation models

## Abstract

**Background:**

Understanding recombination rates is crucial in evolutionary biology, as recombination shapes genetic diversity, natural selection, and adaptation. We investigated recombination rate variation in *Chironomus riparius* across different latitudes, seasons, and experimental treatments using Pool-seq data from five studies and the ReLERNN neural network-based method. We examined its relationship with genetic diversity, GC content, and F_ST_, assessing causality through structural equation modeling.

**Results:**

In natural populations, recombination rates showed no clear latitudinal pattern, likely due to interactions between climate-driven selection, demographic history and regional environmental heterogeneity. However, seasonal variation was evident, with higher recombination rates in autumn than winter, possibly due to temperature-induced plasticity or seasonal bottlenecks. A cold snap in March 2018 triggered a sharp recombination increase, potentially suggesting a stress-induced adaptive response. Across datasets, recombination rates were correlated with genetic diversity and other genomic parameters, with structural equation models (SEMs) indicating that recombination and selection jointly shape patterns of π and differentiation, while relationships with GC content and TEs counts varied among environmental and experimental contexts. In experimental populations, thermal regimes alone had little effect on recombination; instead, adaptation to laboratory conditions and specific stressors drove recombination changes. Exposure to microplastics led to a genome-wide reduction in recombination, likely due to stress-induced DNA repair prioritizing genome integrity, whereas cadmium exposure generally suppressed recombination.

**Conclusions:**

Our results demonstrate that recombination in *C. riparius* is a highly dynamic trait influenced by environmental conditions, selection, and genomic context. By integrating ecological variation, experimental evolution, and multivariate genomic analyses, this study highlights recombination as a context-dependent process that responds to both natural and anthropogenic stressors and interacts with multiple features of genome architecture.

**Supplementary Information:**

The online version contains supplementary material available at 10.1186/s12864-026-12809-5.

## Background

Life’s diversity emerges from the complex interplay between the environment, evolutionary forces and the genomes they shape. Recombination, the physical exchange of genetic material between homologous chromosomes, is a fundamental evolutionary mechanism that generates genetic diversity, facilitates adaptation, and influences genome evolution [1]. By reshuffling alleles and producing novel genetic combinations, recombination modulates natural selection [2, 3] and contributes to the distribution of genetic variation across the genome [4, 5]. Despite being a conserved meiotic process, recombination rates (*rr*) exhibit substantial variation across genomes, species, and populations due to evolutionary, environmental, and stochastic factors [6].

Recombination rate variation is observed at multiple levels. Closely related species display different recombination landscapes due to distinct genome architectures and evolutionary histories [7, 8]. Within species, recombination rates can vary based on genetic background, environmental conditions, and demographic history of the constituent populations [9–11]. Within populations, recombination rates are influenced by both genetic and stochastic factors, including chromosomal variation [12, 13] and sex-specific differences, with females typically exhibiting higher crossover rates than males [14, 15]. Furthermore, recombination is not uniformly distributed along chromosomes; certain genomic regions, known as recombination hotspots, experience higher crossover frequencies. While recombination does not directly impact an individual’s survival, it shapes genetic variation in offspring and influences the evolutionary trajectory of populations, potentially playing an adaptive role [6, 16–19] - in the same way that variation in fitness-related traits does.

A growing body of research highlights the role of biological and environmental factors in driving recombination rate variation. Geographic heterogeneity and latitudinal gradients can shape recombination landscapes by exposing populations to distinct selective pressures and genetic drift regimes [18, 20, 21]. For instance, studies in *Drosophila melanogaster* have revealed regional differences in recombination rates, with local adaptation and demographic history contributing to these patterns [22]. Environmental gradients and ecological clines can also influence recombination, as organisms in fluctuating environments may exhibit elevated recombination rates to enhance adaptive potential [23–25].

Among environmental factors, temperature is a well-documented extrinsic modulator of recombination rates [26–29]. Experimental studies in *D. melanogaster* demonstrate the plasticity of recombination, with increased crossover rates observed in response to temperature fluctuations [30–32]. Beyond temperature, seasonal changes have been shown to impact recombination rates as organisms respond to cyclic environmental stressors. In *D. melanogaster*, crossover rates differ between winter and autumn populations, suggesting that recombination may be influenced by seasonal selection pressures [33]. Theoretical models further suggest that recombination rate modifiers are more likely to persist in fluctuating environments when linked to loci under selection [34]. A substantial body of research has employed experimental setups using non-temperature stressors to investigate recombination, offering critical insights into the mechanisms and factors influencing this fundamental evolutionary process [35–37].

In addition to environmental influences, recombination is correlated with specific genomic features. In honeybees, DNA structure significantly influences recombination frequency, though the mechanisms underlying high recombination rates remain incompletely understood [38]. GC content has been linked to recombination in multiple taxa, including nematodes, *Drosophila*, chickens, and mammals (nematodes and *Drosophila* [39, 40]; *D. melonogaster* [41]; chickens [42]; mouse [43]), though studies in yeast suggest that GC content alone may not always drive recombination [44] or GC content acting as a sole modifier of recombination [45]. Recombination also plays a key role in shaping linkage disequilibrium and nucleotide diversity (π) [3]. Strong correlations between recombination rates and genetic diversity have been documented in *Drosophila* [22, 46–50] and other taxa (butterflies [51]; mice and rabbits [52]; chickens [53]; yeast [54]), though this relationship appears weaker or absent in some plant species [55] and in *D. pseudoobscura* [56].

Another genomic feature associated with recombination is the distribution of transposable elements (TEs; [57–59]. TEs are not randomly dispersed across genomes, and their abundance correlates negatively with recombination rates in *Drosophila*, likely due to stronger purifying selection against TE insertions in high-recombination regions [60–64]. However, this pattern is not universal; in *Caenorhabditis elegans*, recombination rates correlate positively with TE abundance, suggesting lineage-specific variation in the forces governing TE dynamics (RNA-based elements; [65]. Differences in population sizes, selection pressures, and TE families likely contribute to these contrasting patterns [66].

While much of our understanding of recombination comes from model organisms such as *D. melanogaster*, recombination rate variation remains largely unexplored in many non-model species. Furthermore, most studies have focused on spatial variation in recombination, whereas fewer have examined how recombination varies across temporal scales, particularly in response to seasonal or multi-generational environmental changes. Experimental evolution approaches provide a powerful framework to investigate these temporal dynamics, offering insights into how recombination rates evolve under controlled selective pressures. In this study, we conduct an exploratory analysis to investigate recombination rate variation in *Chironomus riparius* using multiple Pool-Seq datasets. Specifically, we aim to examine how recombination rates fluctuate across geographic locations, seasonal changes, and experimental selection regimes. Here, we address the following questions: (i) Does recombination rate variation in *C. riparius* exhibit predictable ecological structure, including latitudinal gradients, seasonal effects, or responses to experimental environmental treatments? (ii) How does recombination rate variation relate to key genomic features, including genetic diversity, GC content, TE abundance and population differentiation (F_ST_)? By integrating these datasets, we seek to provide new insights into the forces shaping recombination rate variation and to expand our understanding of the evolutionary and ecological significance of recombination.

## Materials and methods

### Datasets trimming and re-mapping

**Fig. 1:**
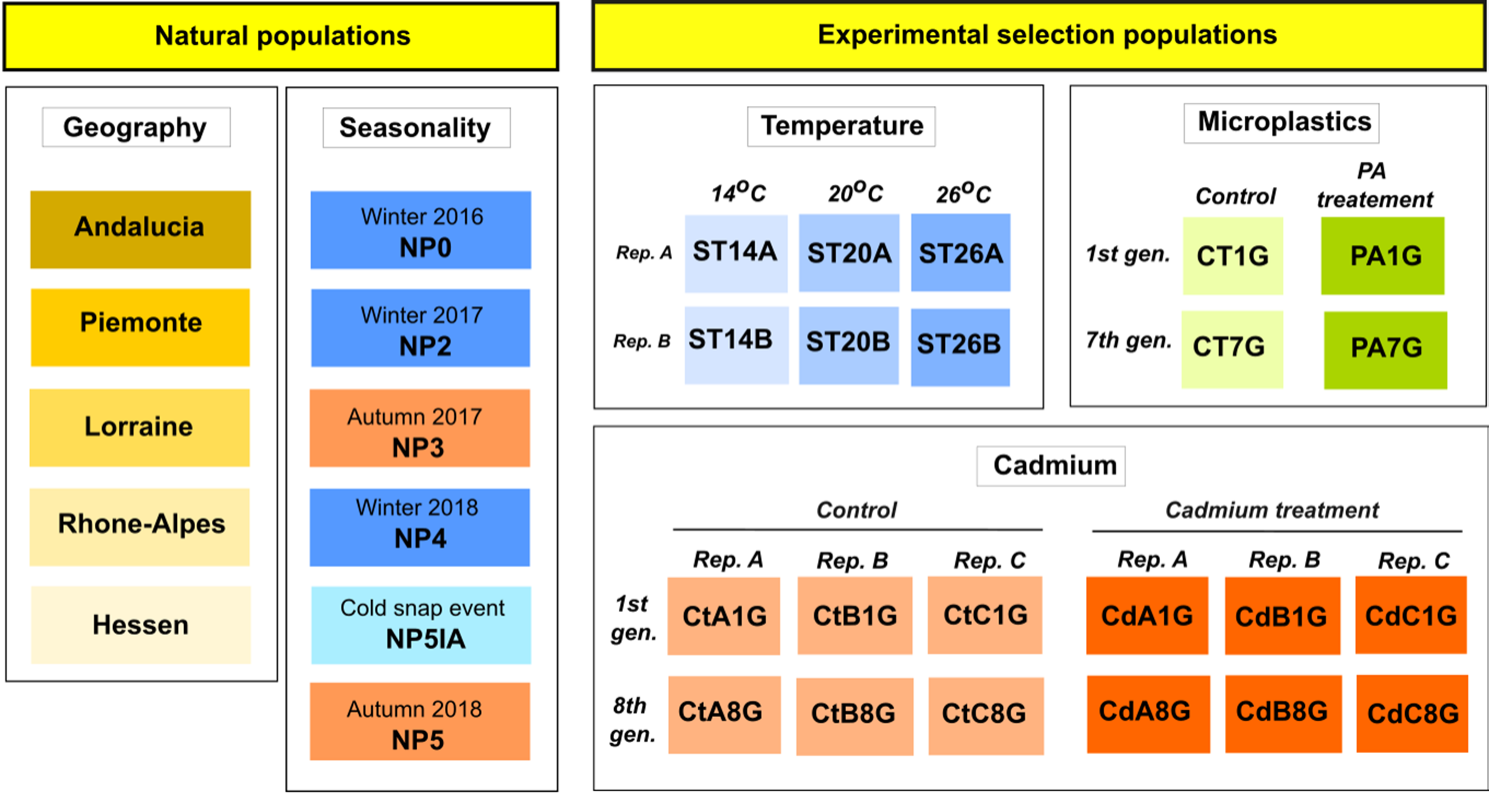
Description of identifiers for pools from geography, seasonality, temperature, microplastics and cadmium datasets. *Rep.* = replicat; *gen.* = generation

We download Pool-seq data from the European Nucleotide Archive (ENA) for 5 published studies investigating different aspects of the evolutionary and molecular ecology of *C. riparius* (Table S1). *C. riparius* is a multivoltine species of non-biting midge that can produce up to 15 generations annually [67] and is widely distributed in the temperate northern hemisphere [68]. Detailed information on the biology of the species, samples collection experimental setup, and raw Pool-seq data pre-processing can be found in the specific publications (Table S1). Two datasets represent natural populations (geography and seasonality), while three datasets derive from experimental populations (temperature selection, microplastics exposure, and cadmium treatment). Identifiers (IDs) for all datasets are provided in Fig. 1.

Among the datasets used in this study, only the dataset from [69] was already trimmed. For the remaining four datasets, we trimmed adapters and low-quality bases from paired reads with Trimmomatic v.0.39 [70] using 8 threads (-t 8) and a phred score of 33. We assessed quality of reads with using FastQC [71]. Clean reads ໿from all five datasets were mapped to the chromosome level reference genome of *C. riparius* [72] with the Burrows–Wheeler Aligner (BWA) mem algorithm [73] using parallel threads (-t 10) and minimum seed length of 30 (-k 30). We used samtools v.1.20 *mpileup* module (with -B and -Q 0, to ensure that no bases are excluded a priori during pileup generation) to combine pools for each dataset independently. We used Popoolation2 [74] v.1.201 script *mpileup2sync.pl* to convert each mpileup file into sync files using a minimum quality of 20 (--min-qual 20). Using the functions *read.sync* and *af* from the R package *poolSeq* [75] we calculated allele frequencies (AF) for each pool keeping only for biallelic loci.

### Recombination rates analyses

In order to estimate recombination rates across the genome we used ReLERNN v.1.0.0 [76], a recurrent neural networks (RNN)-based method for estimating the genomic map of recombination rates per chromosome directly from AF for each pool independently. This method provides a more flexible, efficient, and noise-tolerant (derived from uneven coverage and sequencing errors) alternative to traditional methods, especially for Pool-seq data, where phasing is principally not available. Its machine learning approach allows it to adapt better to diverse evolutionary scenarios. First, we used ReLERNN_SIMULATE to split the input of AF and ran the simulations using a window size of 10 kb, which included simulate training, validation, and test using coalescent simulation program *msprime*. In a second step, ReLERNN_TRAIN used the simulations from the previous step to train a recurrent neural network. We used 250 epochs to train over and 10 validation steps. Following the training, we used the module ReLERNN_PREDICT to predict per-base recombination rates in 10 kb non-overlapping windows across all chromosomes of the genome. Finally, we obtained 95% CI (confidence interval) around each predicted recombination rate with the module ReLERNN_BSCORRECT. For each dataset, we inspected if recombination rates were statistically different with the R package *Bayesian First Aid* [77] function *bayes.t.test* by comparing pairwise differences of pools within each dataset.

### Estimation of genetic diversity and GC content

To estimate genetic diversity as π, we used Popoolation1 v.1.2.2 [74] for each pool. We used the mapped clean reads and samtools *mpileup* function to obtain mpileup files for each pool. For π estimation, we used the *Variance-sliding.pl* function with a *--min-count* of 3, a *--min-coverage* of 15, a *--max-coverage* of 50 and a *--min-covered-fraction* of 0.5. We run all analyses using a *--window-size* and *--step-size* of 1000 to avoid overlapping windows. Because effective population size (*N*_*e*_) can influence genome-wide levels of π and allele frequency dynamics, we note that the datasets analyzed here derive from previously published experimental evolution studies. In those studies, populations were initiated with defined census sizes and monitored to avoid demographic bottlenecks, and *N*_*e*_ was estimated based on allele frequency changes following [75]. *N*_*e*_ was not re-estimated in the present study.

We estimated GC content with a custom Python script (Supplementary material) using a window size of 10 kb. Because the script required the pool in .fasta format, we previously split each .bam file by chromosome and converted them to .fasta format using samtools *view* and samtools *fasta* respectively. To test whether recombination rates is correlated to π or GC content, we used the function *bayes.cor.test* in the R package *Bayesian First Aid* [77] with the default uniform prior on the correlation coefficient (− 1, 1). These correlation analyses were based on the estimation of 10 kb non-overlapping windows of the parameters. While this approach minimizes methodological autocorrelation due to overlapping bins, some spatial autocorrelation among adjacent windows is expected due to underlying genomic linkage. The analyses are intended to capture broad-scale associations rather than to perform formal spatial inference.

### Genetic differentiation

For the experimental datasets and the time series dataset we calculated the genome-wide F_ST_ using Popoolation2 v.1.201 *fst-sliding.pl*. We looked at F_ST_ pairs between the 1st and 7th generation microplastics (PA1G-PA7G) treatment, and the 7th generation of control and microplastics treatment (CT1G-PA7G) in the microplastics dataset. For the Cadmium dataset we looked at F_ST_ pairs between the 1st and 8th generation Cadmium treatment (Cd1G-Cd8G) and the 8th generation control and Cadmium treatment (Ct1G-Cd8G). For the temperature selection dataset we estimated the between the time series NP0 pool (ancestral population for all experimental datasets) and each of the different temperatures treatments and replicates. In addition, we calculated the F_ST_ between NP0 pool and first and last generation for the Cadmium and microplastics dataset and NP0 pool and all treatment and replicates for the temperature dataset to be included in the structure equation model analysis (discussed below).

We investigated the relationship between F_ST_ and significant shifts in recombination rates in the experimental datasets (temperature, microplastics, and cadmium) to assess how recombination rate distributions changed between control and treatment pools over time. This analysis aimed to determine whether genomic regions experiencing substantial recombination rate shifts across generations were associated with regions of high F_ST_, which we interpret as a proxy for selection. We defined regions of high F_ST_ as regions with F_ST_ values above the 95% percentile threshold. We calculated the delta difference (Δ) in recombination rates between the first generation control and treatment groups, as well as between the control and the last generation. For the temperature dataset, Δ was calculated between each treatment and the NP0 pool from the seasonality dataset, representing the ancestral population. To identify genomic regions with substantial shifts in recombination rates, we extracted regions where Δ exceeded the 95% percentile. The corresponding F_ST_ estimates for these regions were then obtained to examine potential associations. Statistical relationships were assessed using the *bayes.cor.test* function from the R package *Bayesian First Aid* [77].

### Estimation of TEs

As required by PoPoolationTE2, v.1.10.03 [78] we first prepared a custom made library a of *C. riparius* specific transposable elements and repeats with RepeatModeller v.2.0 [79] and -engine NCBI (National Center for Biotechnology Information). We used the headers of each of the sequencers in the library to create a TE hierarchy, an additional requirement from PoPoolationTE2, extracting insert name, family and order. We obtained a TE reference genome by combining the TE library and the reference genome. We mapped paired-end sequence reads of each pool from each dataset to the TE refence genome using BWA alignment algorithm *bwasw* [73]. Then we converted the resulting .sam files to bam files with PoPoolationTE2 function se2pe. We generated a mpileup file with all bam files per dataset with PoPoolationTE2 function *ppileup*, with a min quality --map-qual 15. To avoid biased comparison of TE abundance between pools within each dataset, we used the PoPoolationTE2 function *subsampleppileup* with target-coverage 55 to obtain a uniform coverage and thus, homogenize the power to identify TE insertions. In order to estimate population frequency of TE insertions and rearrangements we used the PoPoolationTE2 functions *identifySignatures* and *frequency*. TE insertions were analyzed as total counts per sample, as higher-level TE classification (Class I vs. Class II) was not consistently encoded in the reference annotation used by PoPoolationTE2. Because TE insertions exhibit non-random genomic distributions, the observed associations between total TE abundance and recombination rate should be interpreted as genome-wide trends rather than as evidence for local insertion biases relative to fine-scale recombination landscapes.

### Structural equation models

**Fig. 2:**
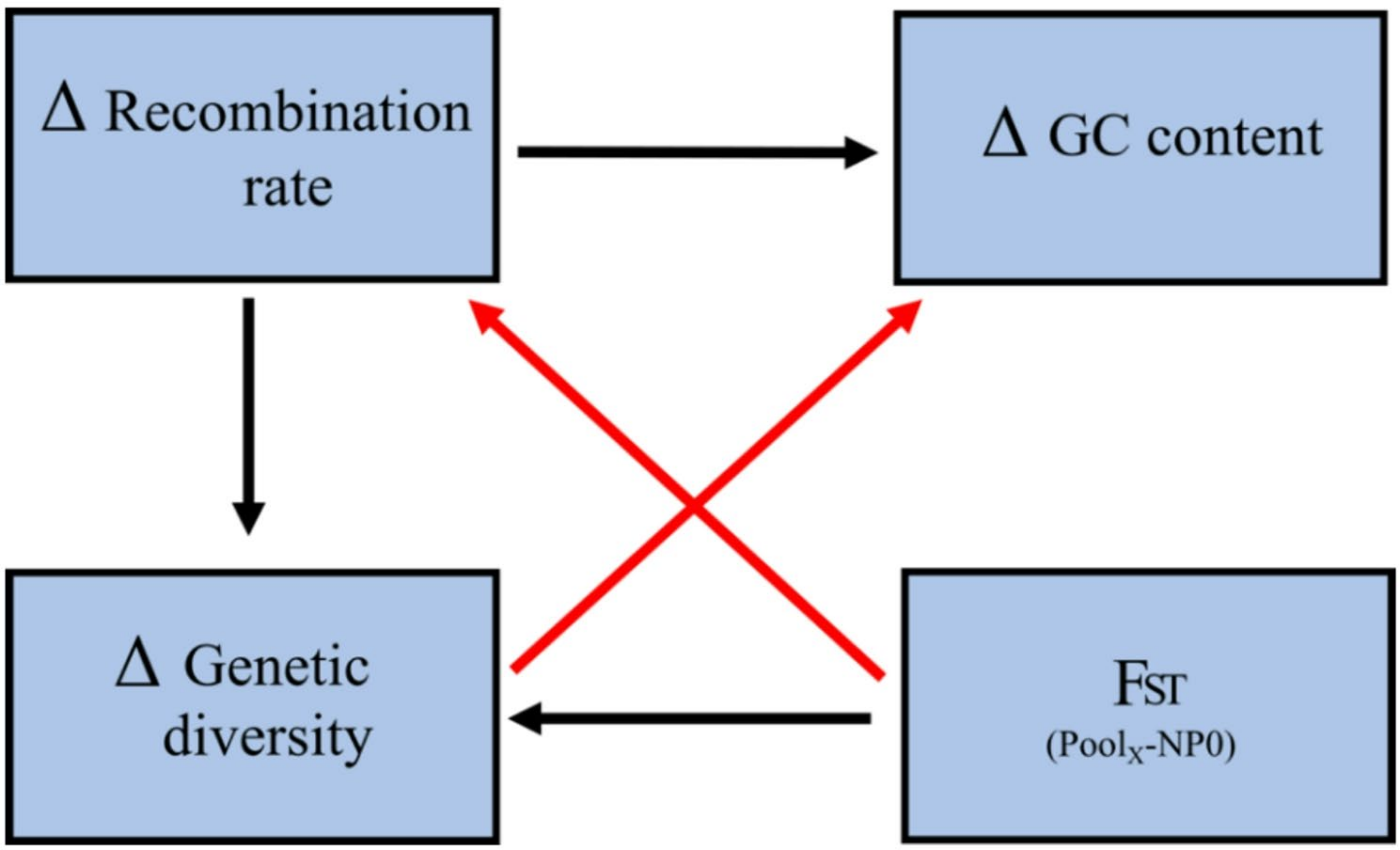
Structural equation model (SEM) of hypothesised causal and covariance relationships tested in this study for the experimental selection datasets: temperature, microplastic and Cadmium. Black arrows indicate a causal relationship and red arrows indicate a covariance relationship. Δ indicates the difference between a particular pool and NP0 pool from seasonality dataset (ancestral pool). F_ST_ corresponds to the estimation between a particular pool and NP0

For the three experimental datasets we tested hypothesized causal relationships among different genomic parameters in order to explore the multivariate relationships among them through a series of structural equation models (SEMs). We included recombination rates, genetic diversity and GC content as delta (Δ) differences between the parameter of a particular pool with the NP0 pool from the time series dataset (ancestral pool). We used the Δ of the parameters to provide insights into how the treatment alters the relationships and dynamics between these parameters, rather than just describing their existing associations. We also include F_ST_ estimations as a pairwise estimation between a pool and the NP0 pool. Causal relationships included the impact of recombination rates and F_ST_ on genetic diversity. We used here F_ST_ as a proxy for selection, which in addition has an impact on genetic diversity. We performed the SEMs using piecewiseSEM v.2.3.0 [80] in R for all experimental datasets. We used established causal and covariance relations as shown in Fig. 2. Model fit and conditional independence claims among nodes for each pool was evaluated using Fisher’s C value, with *p*-values showing statistically significance between model and data.

## Results

### Recombination rates estimation

Mean recombination rates varied across datasets and pools within each dataset. For the geography datasets the Hessen and Rhone-Alpes pools show the highest recombination rates (2.84e-09 ± 3.60E-09 c/bp (centimorgan/base pair) and 2.81e-09 ± 3.26E-09 c/bp) and Andalucia showed the lowest recombination rate (1.32e-09 ± 1.71e-09 c/bp). In the seasonality dataset the NP5 pool (autumn 2018) showed the highest mean recombination rate (4.78e-09 ± 3.68E-09 c/bp) followed by the cold snap pool NP5IA (winter March 2018–4.38e-09 ± 3.21E-09 c/bp), whereas NP4 (winter 2018) showed the lowest (2.16e-09 ± 2.59E-09 c/bp). Mean values across different pools in the selection temperature dataset showed minimal variation. Both replicates of the 20 °C treatment pools showed the lowest and highest recombination rates (ST20A = 5.77e-09 ± 3.63E-09 c/bp and ST20B = 6.92e-09 ± 3.98E-09 c/bp). In the selection microplastic dataset the polyamid (PA) treatment seventh generation (PA7G) pool showed the lowest recombination rate (1.85e-09 ± 2.24E-09 c/bp) and control first generation (CT1G) showed the highest (3.46e-09 ± 3.52E-09 c/bp). Pools under PA treatment showed lower recombination rates as their respective control pool. In the selection cadmium dataset the lowest recombination rates for all replicates were the Cadmium 8th generation treatment. The pools with the highest recombination rate in all replicates were the Cadmium treatment pools of 1st generation (Table 1).

**Table 1:** Summary of recombination rates of all pools; π estimation in 10 kb windows; correlation between recombination rates and π; GC content in 10 kb windows; correlation between recombination rates and GC-content; and number of TEs insertions per pool. Descriptive explanation of datasets and pools IDs can be found in Fig. 1.*Corr*.=correlation; *rr*=recombination rates

Agent		Pool ID’s	rr (c/bp)	pi	Corr. rr - pi	GC	Corr. rr - GC	# TEs
Geography		Andalucia	1.32E-09	0.009	0.27	0.308	NO	57
		Piemonte	2.06E-09	0.01	0.25	0.293	NO	56
		Lorraine	1.72E-09	0.009	0.31	0.31	NO	56
		Rhone-Alpes	2.81E-09	0.011	0.27	0.311	NO	57
		Hessen	2.84E-09	0.009	0.20	0.317	NO	58
Seasonality		NP0	3.07E-10	0.011	0.40	0.32	NO	76
		NP2	2.44E-09	0.002	0.35	0.311	NO	79
		NP3	5.37E-09	0.005	0.33	0.302	NO	83
		NP4	2.16E-09	0.012	0.20	0.315	NO	78
		NP5IA	4.38E-09	3.04E-05	NO	0.31	NO	76
		NP5	4.78E-09	0.011	0.29	0.322	NO	111
Selection	Temperature	ST14A	6.30E-09	0.011	NO	0.317	NO	93
		ST14B	6.33E-09	0.011	moderate (0.15)	0.316	NO	57
		ST20A	5.77E-09	0.011	moderate (0.19)	0.317	NO	57
		ST20B	6.92E-09	0.010	0.21	0.317	NO	64
		ST26A	6.46E-09	0.009	moderate (0.10)	0.321	NO	56
		ST26B	6.48E-09	0.0004	0.34	0.309	NO	61
	Microplastics	CT1G	3.46E-09	0.004	0.40	0.288	NO	218
		PA1G	2.51E-09	0.006	0.21	0.288	NO	224
		CT7G	3.12E-09	0.006	0.58	0.295	low moderate (0.11)	363
		PA7G	1.85E-09	0.008	0.42	0.299	NO	194
	Cadmium	CTA1G	1.22E-09	0.0004	0.25	0.308	NO	305
		CTA8G	1.72E-09	0.002	NO	0.303	NO	251
		CTB1G	2.08E-09	0.0003	0.25	0.297	NO	265
		CTB8G	1.93E-09	0.001	0.37	0.3	NO	263
		CTC1G	8.84E-10	0.0004	0.22	0.295	low moderate (0.1)	230
		CTC8G	8.45E-10	0.001	0.36	0.299	NO	227
		CdA1G	2.95E-09	0.0004	0.28	0.307	NO	231
		CdA8G	8.95E-10	0.001	0.26	0.303	NO	209
		CdB1G	2.19E-09	0.0004	0.24	0.302	low moderate (0.11)	239
		CdB8G	8.96E-10	0.0006	NO	0.299	NO	227
		CdC1G	1.95E-09	0.0004	0.26	0.295	NO	205
		CdC8G	1.74E-09	0.001	0.38	0.304	NO	232

For the geography, selection temperature and microplastics datasets, chromosome 4 in all pools showed the highest recombination rate. In the seasonality dataset, pools NP0 and NP2 showed the highest recombination rates in chromosome 4 and, pools NP3, NP4, NP5IA and NP5 in chromosome 3. Eight out of the 12 pools from the selection Cadmium dataset showed the highest recombination rates in chromosome 4, while the remaining pools showed the highest recombination rates in either chromosome 1, 2 or 3.

Distribution of 10 kb averages recombination rates along the genome showed the decrease of the rates at the start and end of chromosome (Figure S1a-d). The genome-wide distribution of recombination rates in the Hessen pool, compared with experimental datasets, revealed significantly lower recombination rates in the Hessen pool than in the temperature dataset pools. This pattern was not as clearly observed in the microplastics and cadmium dataset pools (Figure S2).

Within each dataset pool pairwise comparisons showed that all observed differences amongst pools were different from zero with the exception of replicates A and B for the 26 °C treatment of the temperature dataset and control first generation and Cadmium first generation of replicate B of the Cadmium dataset (Table 1).

**Fig. 3:**
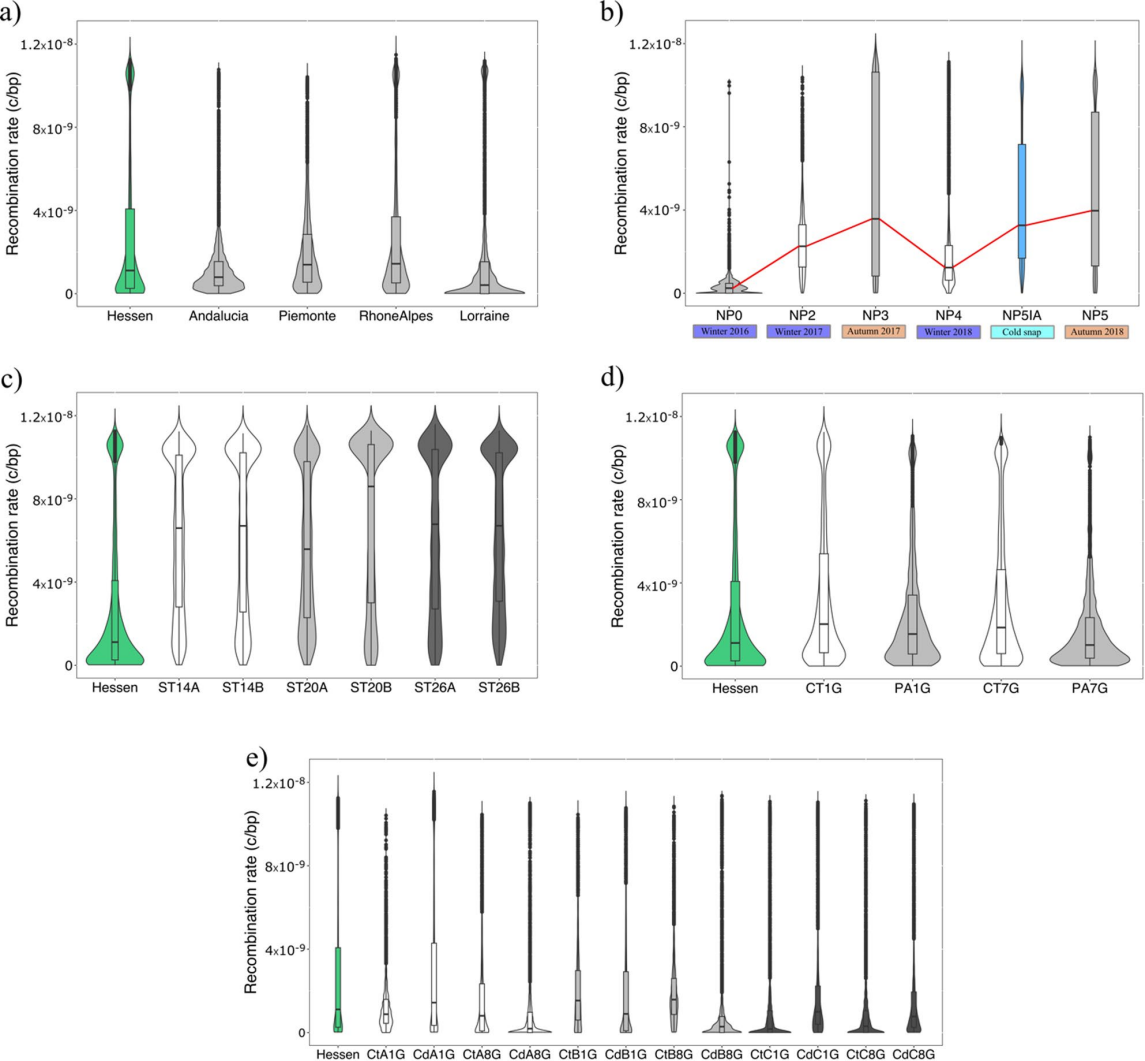
Recombination rates comparison of natural populations datasets: (**a**) geography and (**b**) seasonality; and experimental selection datasets: (**c**) temperature, (**d**) microplastics and (**e**) Cadmium datasets. For the experimental selection datasets populations the ancestral population (Hessen) is shown for comparison

Comparison of Hessen pool from the geography dataset, from which the ancestral source population for the selection temperature, microplastics and Cadmium datasets pools come from, are shown in Fig. 3 and Figure S2.

### Differences in genetic diversity, GC content and TEs across pools

**Fig. 4:**
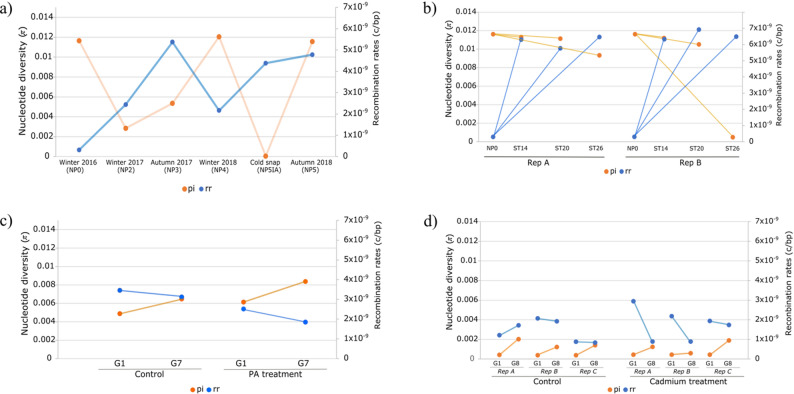
Mean genetic diversity (π; primary Y axis) and mean recombination rates estimates (rr; secondary Y axis) of (**a**) seasonality dataset; (**b**) temperature dataset. Rep= replicate. NP0 = ancestral pool from seasonality dataset (NP0), ST14, ST20 and ST26 refers to the different temperature treatments, 14 °C, 20 °C and 26 °C, respectively; (**c**) microplastics dataset. G1 = generation 1 and G7 = generation 7; and (**d**) Cadmium dataset. Rep= replicate. G1 = generation 1 and G8 = generation 8

The π mean average estimations were generally low for all pools and range from 3.04e-05 in the for the cold snap pool (NP5IA) to 0.012045 from the 2018 winter pool (NP4) from the seasonality dataset (Fig. 4; Table 1). Pairwise differences were statistically significant, however a relevant effect size was only observed between 1st and 7th generation of the PA treatment of the microplastic dataset, between the different temperature treatments in replicate B of the temperature selection dataset and between the control and Cd treatment of 8th generation for all replicates in the Cd dataset.

GC content average estimation range from 0.28899 for the Control 1st generation of the microplastics dataset to 0.322158 for the Autumn 2018 pool (NP5) from the seasonality dataset (Table 1). Pairwise differences were statistically significant, however significant effect size was only observed between 1st and 7th generation of the control and PA treatment of the microplastic dataset, between the 14 °C and 26 °C, and 20 °C, and 26 °C, of both replicates in the temperature selection dataset, between the control and Cd treatment of 1st generation for replicate C in the Cd dataset, and between all pools in the time series dataset.

The abundance of TEs in pools, as measured by the count of TEs in the genomes, range from 56 in several pools (from geography and selection temperature datasets) to 363 (in microplastics dataset). Furthermore, average number of TEs of the microplastics and Cadmium datasets was 3.5 times higher than average number of TEs of the climate, seasonality and temperature selection dataset (Table 1).

### Correlation between recombination rates and different genomic parameters

Bayesian pair test showed generally a strong positive association between recombination rates and π for all pools except for the cold snap pool from the seasonality dataset (NP5IA), replicate A of the 14 °C treatment in the temperature dataset, and control replicate A 8th generation and Cd treatment replicate B 8th generation from the Cadmium dataset (Table 1).

Bayesian pair tests also showed no positive association between recombination rates and GC content for all pools except for low moderate correlation for pools control 7th generation in microplastics dataset and, control replicate C 1st generation and Cd treatment replicate B 1st generation in the Cadmium dataset (Table 1).

**Fig. 5:**
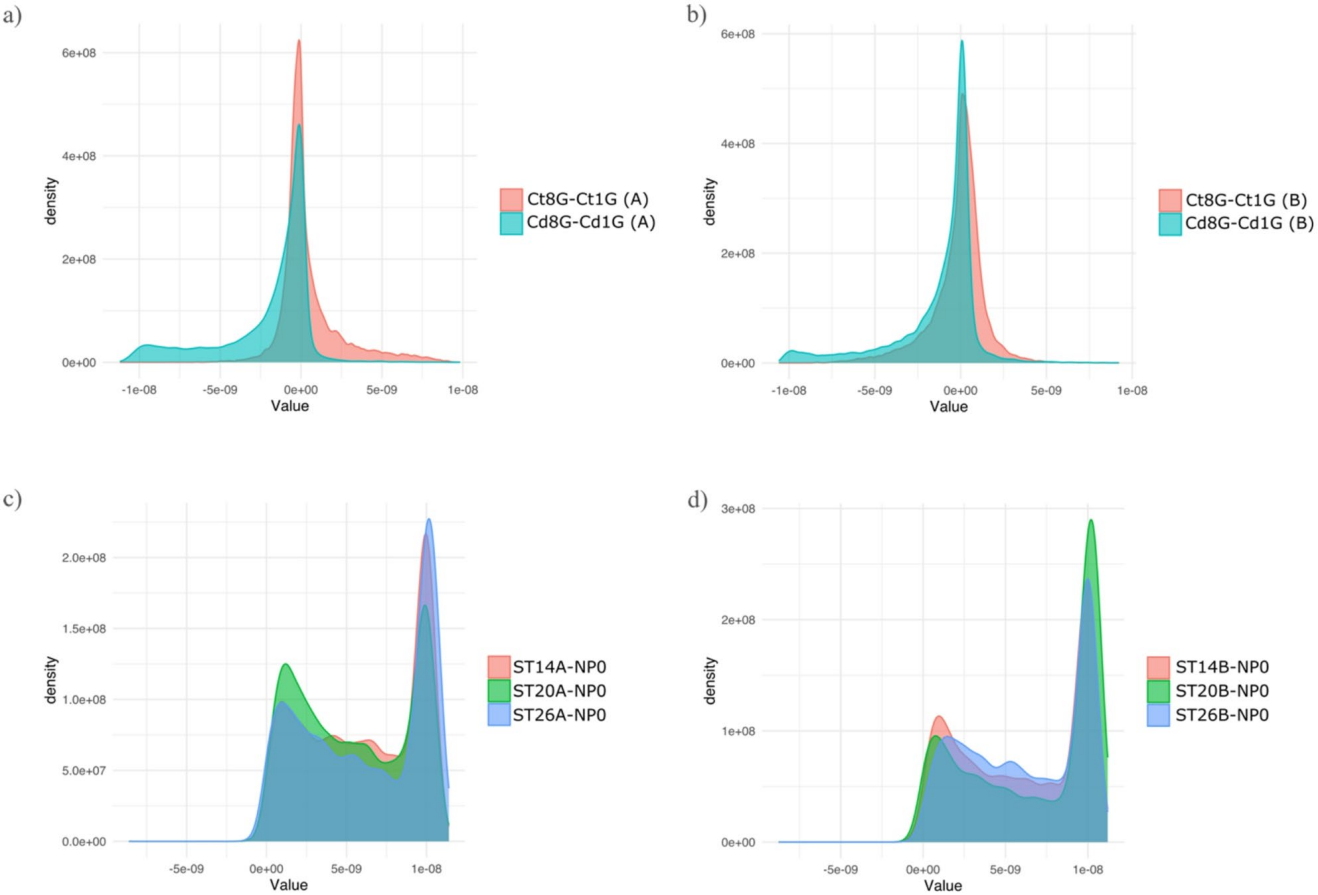
Plots showing the distribution of difference (Δ) in recombination rates between: (**a**) 8th and 1st generation for control (pale red) and Cadmium (cyan) pools replicate A; (**b**) 8th and 1st generation for control (pale red) and Cadmium (cyan) pools replicate B; (**c**) temperature treatments and ancestral pool from seasonality dataset (NP0) in replicate A, temperature dataset; and (**d**) temperature treatments and ancestral NP0 in replicate B

We also identified genomic regions for which the Δ in recombination rates showed a skewed distributions in replicate A and B of the Cadmium dataset and, replicate A and B of the temperature dataset (Fig. 5). In particular, the distribution the Δ in recombination rates for the A and B of the Cadmium treatment extends more into negative values compared to the control Δ distribution (Fig. 5a-b), implying that in some genomic regions, recombination rates decreased more under Cadmium treatment than in the control. Replicate C of the Cadmium dataset and pools from the microplastics datasets did not present this pattern (Figure S3).

We found positive correlation between regions with high Δ in recombination rates and their corresponding F_ST_ estimates for those regions for A and B of the Cadmium treatment (ρ = 0.10 and 0.082, respectively; Figure S4a). 95% HDI (highest density interval) for both correlations do not include zero. However, we found no correlation for all temperature treatments in both replicates A and B, and all 95% HDI did indeed include zero (Figure S4b).

### Structural equation models

In general, the SEM model showed to be a good fit for the majority of the pools analysed. In the temperature dataset Fisher’s C showed that the model explained the data well and no other variables connections are missing, except for the replicate A 20 °C treatment pool, where a relationship between GC content and F_ST_ is proposed with moderate significance (***p < 0.001). Causal relationship between recombination rates and genetic diversity and F_ST_ with genetic diversity are statistically significant for all pools except for replicate A 14 °C treatment and replicate B 26 °C treatment, respectively. Causal relationship between recombination rates and GC content is only statistically significant for replicate A 26 °C treatment and replicate B 14 °C treatment (Table S2). We also detected a reduction in the estimates of the covariance between F_ST_ and recombination rate as temperature increased from 14 °C to 26 °C. (Figure S5a). In the microplastics dataset Fisher’s C showed that the model explained well the data and no other variables connections were missing. All hypothesized causal relationships are statistically supported with high support (***p = 0; Figure S5b). Covariance relationships are only supported for control 7G pool between F_ST_ and recombination rates with moderate support (*p < 0.01). In the replicate A of the Cadmium dataset all hypothesized causal and covariance relationships were statistically supported with high support (***p = 0) for both pools except for the covariance between F_ST_ and recombination rates in the PA treatment 8th generation (Figure S5c). In addition, an additional missing relationship is proposed between GC content and F_ST_ with high statical support (***p = 0; Figure S5c). In the replicate B control and PA treatment for both 8th generations of the Cadmium dataset causal relationships are highly supported except for the relationship between recombination rates and GC content (Figure S5c). In addition, covariance relationships are supported except for the π and GC content in PA treatment 8th generation (Figure S5c). In replicate C, results were only available for control 8th generation, where causal and covariance relationships were highly supported except for causal relationship between recombination rates and GC content, and the covariance relationship between π and GC content (Figure S5c). In addition, an additional missing relationship is proposed between GC content and F_ST_ with low statical support (*p < 0.01; Figure S5c).

## Discussion

Understanding recombination rates is crucial in evolutionary biology, as this process not only drives genetic diversity but also shapes the patterns of natural selection and adaptation across populations. In this study we showed how recombination rates for *C. riparius* vary across a latitudinal range, seasons and under different experimental treatments. As anticipated, our findings reveal substantial variability in recombination rates across the datasets analyzed.

### Variation of recombination rates in natural populations

The five populations represented in the geographic dataset span a broad range of environmental and climatological conditions, reflecting the heterogeneity of regions sampled, from southern to central Europe. Previous works on natural populations have linked diversity in climatic factors and latitudinal cline to variation in recombination rates [18, 23]. The selective pressures derived from climate variation changes continuously, as can be expected for the evolutionary response of populations along these gradients. Therefore, and taking the heterogeneity of regions sampled, it is not surprising that a clear pattern cannot be observed from our data on the relationship of global recombination rates and latitude (Fig. 3a). Work by [69] demonstrated that local climate conditions exert strong selection pressures on the same populations we studied, driving climate adaptation. A study on two ecological (໿xeric vs. sub-alpine) and geographical separated populations of *D. pseudoobscura* discuss the role of natural selection explaining the differences in recombination rates (as accounted by cross-over rate) of the populations [18]. One explanation they offered is that higher temperatures in one of the populations could have caused the increased recombination rates, which is in line with the fact that recombination rates is known to be plastic with regard of ambient temperature [81]. Pettrich & Waldvogel (unpublished observations) recently used whole-genome sequencing (WGS) data from the same five geographically distant populations analyzed in this study and reconstructed genome-wide recombination landscapes, highlighting the potential influence of a transposable element – the *Cla*-element – on recombination rate variation. They found reduced recombination in annotated centromere regions and could find the tendency of the element to occur in association with lower recombination rates. In their analysis, Lorraine, Rhone-Alpes, and Piemonte populations exhibited higher intrapopulation variability given in ρ. Our findings indicate greater recombination rate variation in the Rhone-Alpes, Piemonte, and Hessen populations (Fig. 3a). Furthermore, the variability in Rhone-Alpes and Piemonte align with the other study, while we find different variability for Lorraine. While both studies focused on the same natural populations, we here used different metrics, as ρ is a scaled measured of r (ρ = 4*N*_e_*r), and as such can be directly influenced by *N*_*e*_. The ancient *N*_*e*_ for Lorraine is estimated to be lower than the other populations [72] which could lead to more variability in ρ, also considering effects caused by differences between WGS and PoolSeq data [82].

Although much research has focused on how recombination varies across spatial environmental gradients, temporal variation—particularly across seasons—remains relatively underexplored. Our data reveal a subtle but consistent seasonal fluctuation in genome-wide recombination rates in *C. riparius* (Fig. 3b). Specifically, autumn pool samples (NP3 and NP5) show generally higher recombination rates compared to those collected in winter (NP0, NP2, and NP4). One explanation is that warmer autumn temperatures promote higher recombination rates, while colder winter conditions might physiologically suppress recombination. This pattern aligns with observations in *D. melanogaster*, where recombination is both genetically and plastically reduced under lower temperatures [30]. Additionally [33], found seasonal variation in crossover rates and interference in *D. melanogaster*, suggesting recombination can be shaped by indirect selection driven by seasonal environmental stresses. However, beyond immediate environmental effects, differences in generational turnover between seasons may also play a critical role. In *C. riparius*, generation times are temperature-dependent, with rapid turnover during warmer months (spring and summer) and a developmental pause or zero turnover during winter. Thus, autumn populations are the result of several overlapping generations accumulating over the productive summer, potentially amplifying recombination signatures through repeated meiosis. In contrast, winter samples reflect only a single or even arrested generation, leading to relatively reduced signals of recombination. This generation-count bias offers a compelling explanation that is independent of environmental plasticity. This may be further compounded by reduced *N*_*e*_ in winter due to ecological bottlenecks, which would constrain the recombination signal both through demographic contraction and selection favoring well-adapted genotypes. Taken together, the observed seasonality in recombination rates likely reflects a complex interplay of ecological, developmental, and evolutionary processes, where both environmental plasticity and life-history dynamics influence genome-wide recombination patterns.

Furthermore, we observed that the cold snap of March 2018 (NP5IA) led to a noticeable increase in recombination rates within a short period of time compared to winter 2018 (NP4) (Fig. 3b). This appears as a stress-induced response to the sudden drop in temperatures. This plastic response may help populations rapidly generate new allele combinations in response to selection pressures. Indeed [83], demonstrated that this cold snap resulted in selection for specific polygenic traits to enhance adaptation to sudden environmental changes. Consistent with this, we detected a drastic reduction in genetic diversity in NP5IA, supporting the role of selection in shaping the observed patterns (Table 1). Since the cold snap likely occurred within a single generation, with no new recombination events taking place, selection may have directly influenced recombination rate estimates. Alternatively, it is possible that only individuals with higher recombination rates in previous generations survived, as suggested by our results.

### Experimental selection impact on recombination rates

Our results reveal a complex pattern of recombination rate variation across experimental treatments in the temperature dataset, with distinct but opposing trends observed between the two replicates. However, the different thermal regimes do not seem to have a high impact on recombination rates (Fig. 3c). In replicate A, recombination rates followed a subtle U-shaped pattern across increasing temperature treatments, a pattern observed in *D. melanongaster* [84], while in replicate B, an inverted U-shaped pattern emerged. Notably, we established that these patterns are not driven by temperature regimes themselves but rather by adaptation to laboratory conditions. Importantly, previous work indicates that these trajectories are primarily driven by adaptation to laboratory conditions rather than by the thermal regimes themselves [85]. Despite these contrasting recombination trajectories, both replicates exhibited a consistent decline in genetic diversity.

The opposing recombination trends between replicates suggest that selection under laboratory conditions does not impose a uniform effect on recombination across populations but instead interacts with pre-existing genetic variation and stochastic evolutionary processes. One potential explanation is that recombination rate variation is influenced by genetic background differences between the replicates. If different initial standing genetic variation affects recombination modifiers, selection could drive distinct trajectories in each population. Additionally, recombination itself may evolve as a response to laboratory adaptation, with selection favoring different recombination dynamics depending on the genomic architecture of each replicate.

The concurrent decrease in genetic diversity across all samples suggests that selection has played a dominant role in shaping the genomic landscape, leading to the loss of allelic variation. This supports the hypothesis that adaptation to laboratory conditions has imposed strong selection pressures, reducing neutral diversity while allowing beneficial alleles to rise in frequency.

Across all temperature-selected populations, we identified genomic regions with persistently elevated recombination rates, independent of thermal treatment. Given evidence that selection was driven by laboratory adaptation rather than temperature [85], these regions likely reflect selection for increased recombination in specific genomic contexts. If higher recombination accelerates the assembly of beneficial allelic combinations, selection may favor recombination-enhancing alleles in these regions, leading to stable recombination hotspots even after extended adaptation (22, 44, and 65 generations at 14 °C, 20 °C, and 26 °C, respectively).

Interestingly, we found no correlation between regions showing large changes in recombination rate (Δ recombination relative to the ancestral population) and F_ST_ estimates (Figure S4b). This suggests that recombination rate changes are not directly associated with strong allele frequency differentiation between treatment and ancestral populations. However, interpreting this lack of correlation requires caution due to potential technical and biological caveats. Specifically, regions of high F_ST_ often coincide with areas of reduced π, consistent with selective sweeps, which limits the detectability of recombination because inference methods rely on segregating variation. As a result, recombination may be underestimated in sweep regions, a limitation documented in other systems such as vertebrate breeding populations, where low genetic diversity hampers the tracking of recombination events across generations. Thus, the apparent lack of association between recombination changes and F_ST_ likely reflects both biological decoupling and analytical constraints. Selection may still favor increased recombination at specific loci without generating strong F_ST_ signals, particularly when adaptation proceeds from standing genetic variation.

Regarding changes of recombination rates in relation to non-temperature stressors [86, 87], our study shows that exposure to both microplastics and Cadmium, impacts recombination rates. As observed for the temperature selection dataset, there is a significant increase in recombination rates in the microplastics datasets in comparison to the ancestral population from Hessen (Fig. 3d). In the microplastics dataset, recombination rates were significantly reduced relative to controls, with the effect becoming stronger by the seventh generation (Fig. 3d). Contrary to expectations, microplastic exposure did not induce recombination increases at either generation. One explanation is that stress-induced DNA damage favors rapid repair mechanisms prioritizing genome integrity over genetic reshuffling, potentially shifting repair toward non-homologous end joining (NHEJ) rather than meiotic recombination [88–91]. Microplastic exposure has been linked to oxidative stress and selection on stress-response pathways [92, 93], consistent with enrichment of detoxification and immune-related genes reported in other studies [69, 94–96]. Alternatively, reduced recombination may preserve advantageous allelic combinations under stable or stressful conditions, preventing the breakup of well-adapted genotypes [97–99]. This effect may be amplified in laboratory populations with long-term pre-adaptation to controlled environments. A third possibility is indirect selection on recombination through linkage or epistatic interactions with loci under selection for stress tolerance [19].

Despite reduced recombination, π increased over time in both control and microplastic-treated populations (Fig. 4), indicating that recombination is not the primary driver of increased variation. Instead, elevated mutation rates likely contribute to this pattern. Environmental stressors have been shown to induce high mutation rates in *C. riparius* within a few generations [100, 101], and metal exposure increases mutation rates in *Daphnia pulex* through deletions and duplications [102]. A similar mechanism may be at play in our study. Microplastic-induced oxidative stress may elevate reactive oxygen species (ROS), leading to DNA lesions such as 8-oxoguanine that increase point mutations and indels [103]. If such mutations are incompletely repaired or selectively tolerated, they can increase genetic diversity despite reduced recombination.

Cadmium exposure generally reduced recombination rates relative to controls, with few replicate-specific exceptions (Fig. 3e). This pattern is consistent with stress-induced recombination suppression, potentially mediated by shifts toward NHEJ, as observed for microplastics. The exceptions observed in replicate A (1st generation) and replicate C (both generations) suggest that genetic background, stochasticity, or differing selective pressures modulate recombination responses at the population level. Overall, the reduction in recombination under Cadmium aligns with previous studies showing that toxicants promote conservative genomic architectures, potentially limiting genetic reshuffling and affecting long-term adaptive potential ([35] - DDT; [ dichlorodiphenyltrichloroethane; [104] ] - atrazine; [105–108] - Cadmium). Genetic diversity responses to Cadmium varied across replicates and generations. While diversity was similar between treatments in the first generation, divergence emerged by the eighth generation, with higher diversity in control populations for replicates A and B but higher diversity under Cadmium in replicate C. This heterogeneity indicates that the balance among mutation, recombination, selection, and drift differs across populations. In replicates A and B, large recombination changes between generations correlated positively with F_ST_ from the ancestral population (Fig. 5), suggesting that recombination suppression may reinforce divergence. In contrast, replicate C showed no such association, implying that alternative mechanisms, such as mutation-driven adaptation, might play a larger role.

Finally, both Cadmium and microplastic datasets exhibited markedly elevated TE counts in both control and exposed populations. This pattern likely reflects prolonged laboratory maintenance and reduced effective population sizes rather than direct toxicant effects alone. Although genotoxic stress can disrupt TE silencing pathways (e.g., DNA methylation, piRNA; [60, 109]), the absence of consistent differences between treatments suggests that relaxed purifying selection and genetic drift in long-term laboratory populations facilitate TE accumulation. Over multiple generations, reduced efficacy of TE suppression can lead to gradual TE expansion, highlighting the importance of experimental history and population demography in shaping TE dynamics.

### Recombination dynamics and its causal relationships

The SEMs across the three experimental datasets revealed both shared and dataset-specific causal relationships among recombination rates, genetic diversity, GC content, and F_ST_. Overall, the models highlight the central role of recombination and selection in shaping genome-wide diversity, while also emphasizing that the strength and structure of these relationships depend on environmental context and experimental history.

In the temperature dataset, our findings indicate as expected that recombination rates and F_ST_ exert significant causal effects on genetic diversity in most pools, suggesting that changes in recombination frequency and selection pressures shape genome-wide genetic variability. However, the absence of statistically significant relationships in specific treatments, such as the 14 °C in replicate A and the 26 °C in replicate B, implies that the effects of temperature on these parameters may be context-dependent, potentially influenced by additional factors such as selection pressures derived from the lab conditions (Figure S5a). Notably, covariance between F_ST_ and recombination rates declined with increasing temperature. Given that temperature itself does not directly alter overall recombination rates, this pattern is more plausibly explained by differences in the number of generations exposed to each thermal regime, with stronger associations observed after fewer generations (22 generations at 14 °C) and diminishing after prolonged exposure (65 generations at 26 °C).

In the Cadmium dataset, results varied across replicates, reflecting complex interactions between heavy metal stress and genomic responses. Replicate A showed strong statistical support for all hypothesized causal and covariance relationships, indicating that recombination rates, F_ST_, and genetic diversity were tightly interconnected under Cadmium exposure. The emergence of an additional causal link between GC content and F_ST_ suggests that Cadmium exposure may influence nucleotide composition, potentially through biased mutation or DNA repair processes. In replicate B, most causal relationships were retained, but the recombination–GC content link was not supported, indicating that this association can be disrupted under metal stress. In replicate C, analyses were limited to the control eighth generation, where most hypothesized relationships were supported except for the recombination-GC content causal link and the covariance between genetic diversity and GC content. The presence of a weak additional GC content–F_ST_ relationship further suggests stress-related shifts in nucleotide composition. Collectively, these replicate-specific differences indicate that while recombination and selection consistently shape genetic diversity, the detailed structure of their interactions depends on lineage-specific responses and stochastic effects, underscoring the importance of multi-replicate designs when assessing genomic adaptation to environmental stress.

The microplastics dataset exhibited a more consistent pattern, with strong support for all hypothesized causal relationships. This uniformity suggests that microplastic exposure induces stable and predictable effects on recombination rates, genetic diversity, and GC content. The weak support for covariance between F_ST_ and recombination rates, detected only in the control 7G pool (Figure S5c), indicates that microplastic effects are primarily driven by direct causal pathways rather than co-variation. Given previous associations between microplastics, oxidative stress, and DNA damage, the observed consistency may reflect stress-induced genomic instability influencing recombination dynamics and downstream diversity patterns. Compared to temperature, microplastics may therefore impose stronger and more uniform selective pressures, leading to reproducible genomic responses across replicates. However, as the microplastics dataset spans fewer generations than the temperature experiment, it remains possible that selection initially targets other traits before exerting stronger effects on recombination rates.

## Conclusions

Recombination rates varied substantially among natural populations, suggesting that local adaptation and environmental heterogeneity play a role in shaping recombination landscapes. While a clear latitudinal pattern was not evident, differences in recombination rates among sampling locations may reflect adaptation to regional conditions. We also detected subtle seasonal variation in recombination rates, with autumn populations exhibiting higher rates than winter populations, indicating that recombination can respond to temporal environmental fluctuations. Furthermore, our experimental evolution data revealed that both microplastic and cadmium exposure can influence recombination rates, potentially as a consequence of selection favoring specific genetic combinations. These findings highlight the dynamic nature of recombination and its responsiveness to a range of evolutionary and environmental factors over generations. Our study contributes to a growing body of evidence demonstrating that recombination rate variation is a complex temporally variable in the short-term phenomenon influenced by multiple interacting factors, including genomic features, environmental conditions, and selective pressures. Further research is needed to fully elucidate the specific mechanisms driving recombination rate variation in natural populations and to explore the long-term evolutionary consequences of altered recombination rates in changing environments.

## Supplementary Information

Supplementary Material 1

## Data Availability

Raw data used in this study can be found under ENA project numbers: PRJEB19848, PRJEB35534, PRJEB32795, PRJEB48137 and PRJEB90147.

## Abbreviations

AF: Allele frequency
c/bp: Centimorgan/base pair
CI: Confidence interval
DDT: Dichlorodiphenyltrichloroethane
ENA: European nucleotide archive
IDs: Identifiers
F_ST_: Fixation index
GO: Gene ontology terms
HDI: Highest density interval
NCBI: National Center for Biotechnology Information
N_e_: Effective population size
NHEJ: Non-homologous end joining
PA: Polyamide
ROS: Reactive oxygen species
rr: Recombination rate
SEMs: Structural equation models
TE: Transposable elements
WGS: Whole-genome sequencing
