# Supplementary material for: Influence of geography, seasonality and experimental selection on Chironomus riparius recombination rates
Source: BMC Genomics. 2026 Apr 15;27:393. doi: 10.1186/s12864-026-12809-5 (PMC13094081; doi:10.1186/s12864-026-12809-5)
Supplement: Supplementary file 1 — Supplementary Material 1 [file 12864_2026_12809_MOESM1_ESM.docx]

**Influence of geography, seasonality and experimental selection on *Chironomus riparius* recombination rates**

**María Esther Nieto-Blázquez^1^*, Cosima Caliendo^2^, Laura C. Pettrich^3^, Ann-Marie Waldvogel^4^ and Markus Pfenninger^1,5^**

*^1^Department of Molecular Ecology, Senckenberg Biodiversity and Climate Research Centre, Frankfurt am Main, Germany*

*﻿^2^Institute of Human Genetics; University Medical Center; Johannes Gutenberg University Mainz, Staudinger Weg, Mainz, Germany*

*^3^Institute of Zoology, University of Cologne, Cologne, Germany*

*^4^School of Life Sciences, Technical University of Munich, Limnological Research Station, Hofmark, Iffeldorf, Germany*

*﻿^5^Institute for Molecular and Organismic Evolution, Johannes Gutenberg University, Johann-Joachim-Becher-Weg, Mainz, Germany*

**Supplementary Material and Methods**

**Table S1**. Datasets downloaded from ENA for the estimation of recombination rates.

|  | **Factor** | **Agent(s)** | **ID name for current study** | **Number of pools** | **Publication** | **ENA accession number** |
| --- | --- | --- | --- | --- | --- | --- |
| Natural populations | Geography | Climate, history and local adaptation | Geography | 5 | Waldvogel et al. (2018) | PRJEB19848 |
|  | Seasonality | Seasonal and non-seasonal factors | Seasonality | 6 | Pfenninger & Foucault (2020) | PRJEB35534 |
| Experimental populations | Selection | Lab conditions | Temperature | 6 (3 treatment x 2 replicates) | Pfenninger & Foucault (2019) | PRJEB32795 |
|  |  | Lab conditions | Microplastics | 4 (control and treatment before and after) | Khosrovyan et al. (2022) | PRJEB90147 |
|  |  | Lab conditions | Cadmium | 12 (3 replicates; control and treatment before and after) | Doria et al. (2022) | PRJEB48137 |

**Extraction of GC content.** Python script used to extract GC content from each pool using 10kb windows.

##############################################################

#gets the GC content in windows specified in the infile

from Bio.Seq import Seq

from Bio import SeqIO

import csv

#prepare output file

outfname = "CT7G_chr4_GC_10Kwindows.out"

outf = open(outfname, "w")

outf.write("Scaffold\tGC\n")

scaffID = []

start = []

end = []

#the infile with the start and end positions of 10K chunks per scaffold

infile = "chr4_windows.txt"

outf = open(outfname, "w")

with open(infile) as to_read:

reader = csv.reader(to_read, delimiter = "\t")

for row in reader:

#print ("read row")

scaffID.append(row[0])

start.append(int(row[1]))

end.append(int(row[2]))

for seq_record in SeqIO.parse("CT7G_Chr4.EDITED.fasta", "fasta"):

scaff = seq_record.id

for i in range (0,len(scaffID)):

if scaffID[i] == scaff:

seq = seq_record.seq[start[i]:end[i]]

#determine base composition

freqGC = (float(seq.count("G")) + float(seq.count("C"))) / 10000

print(scaffID[i], " ", start[i], " ", end[i], " ",freqGC)

outf.write(str(scaffID[i]) + "\t" + str(start[i]) + "\t" + str(end[i]) + "\t" + str(freqGC) + "\n")

outf.close()

################################################################

**Supplementary Results**

**Figure S1**. Genome-wide distribution of 10kb recombination rates along four chromosomes from the a) geography, b) seasonality, c) temperature, d) microplastic and e) Cadmium datasets (Mb=million bases).

1. Geography


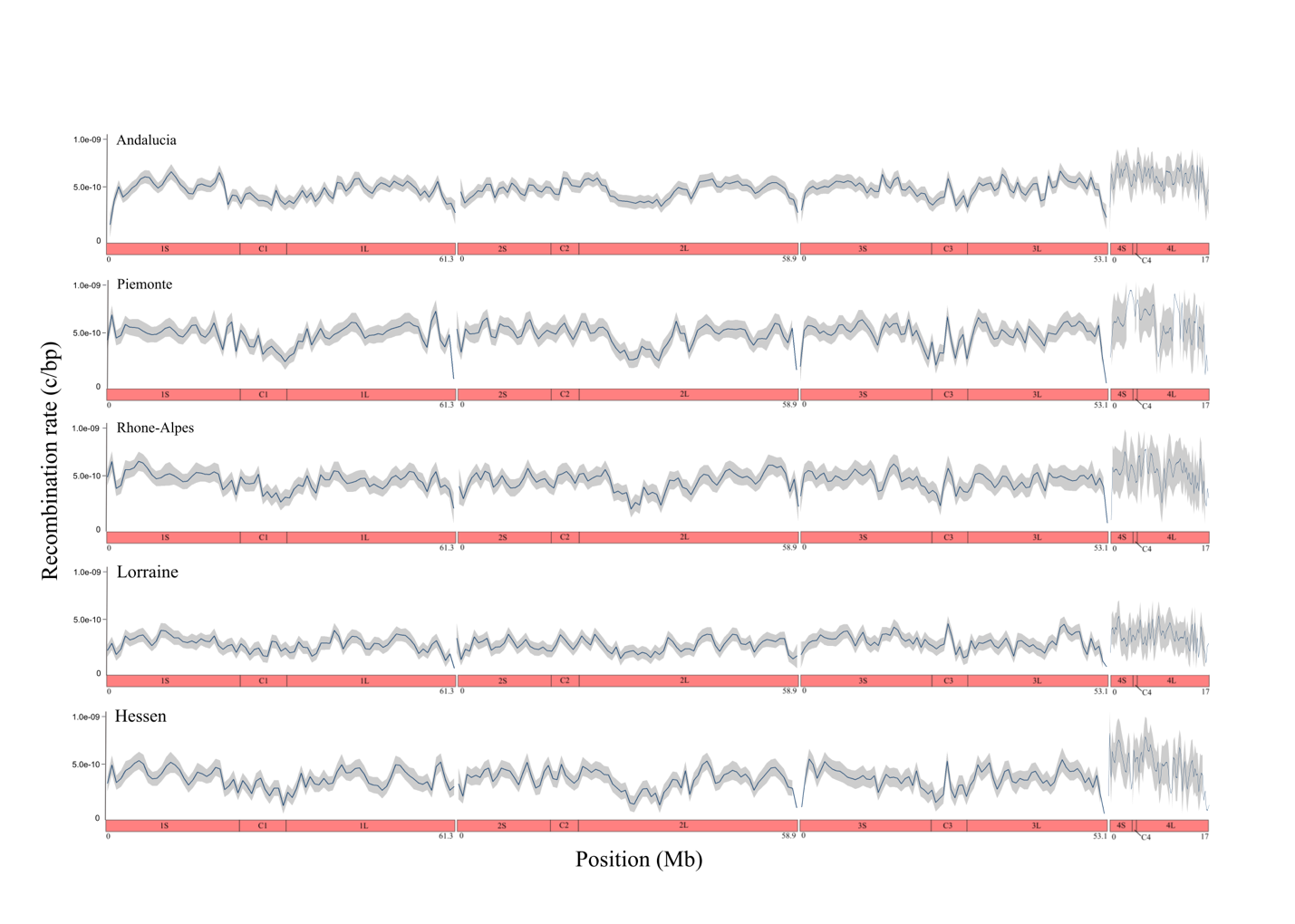


1. Seasonality


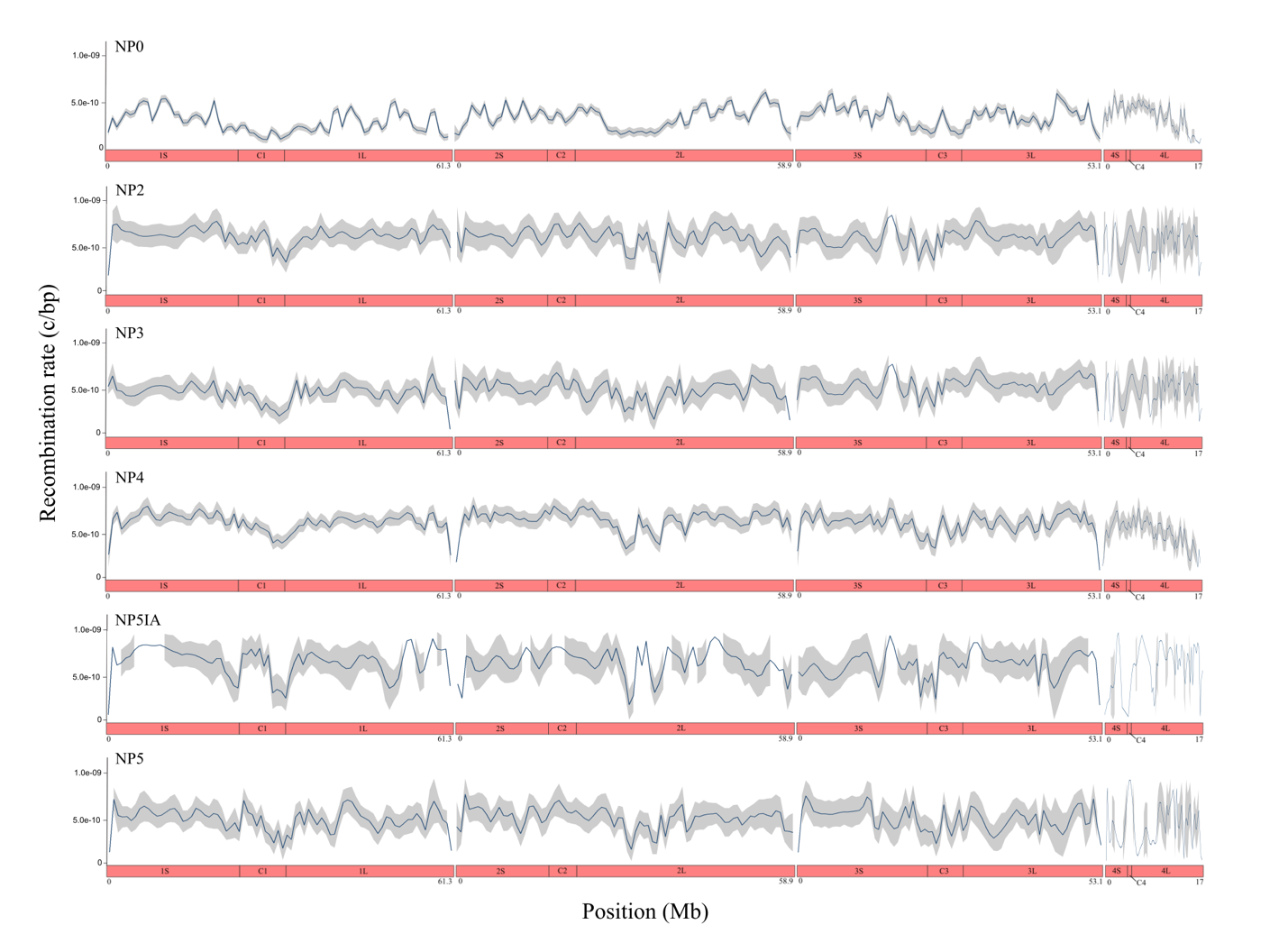


1. Temperature


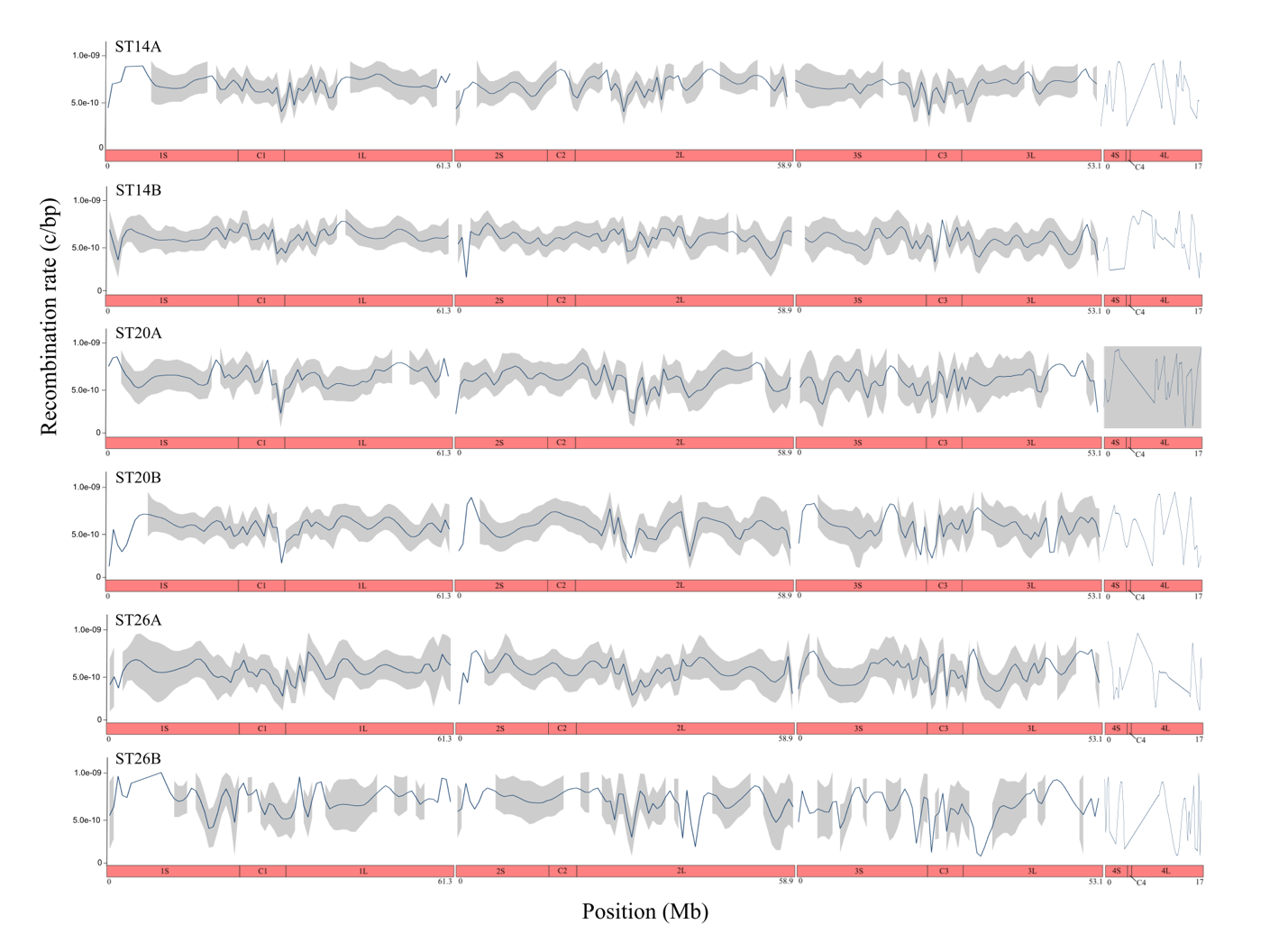


1. Microplastics


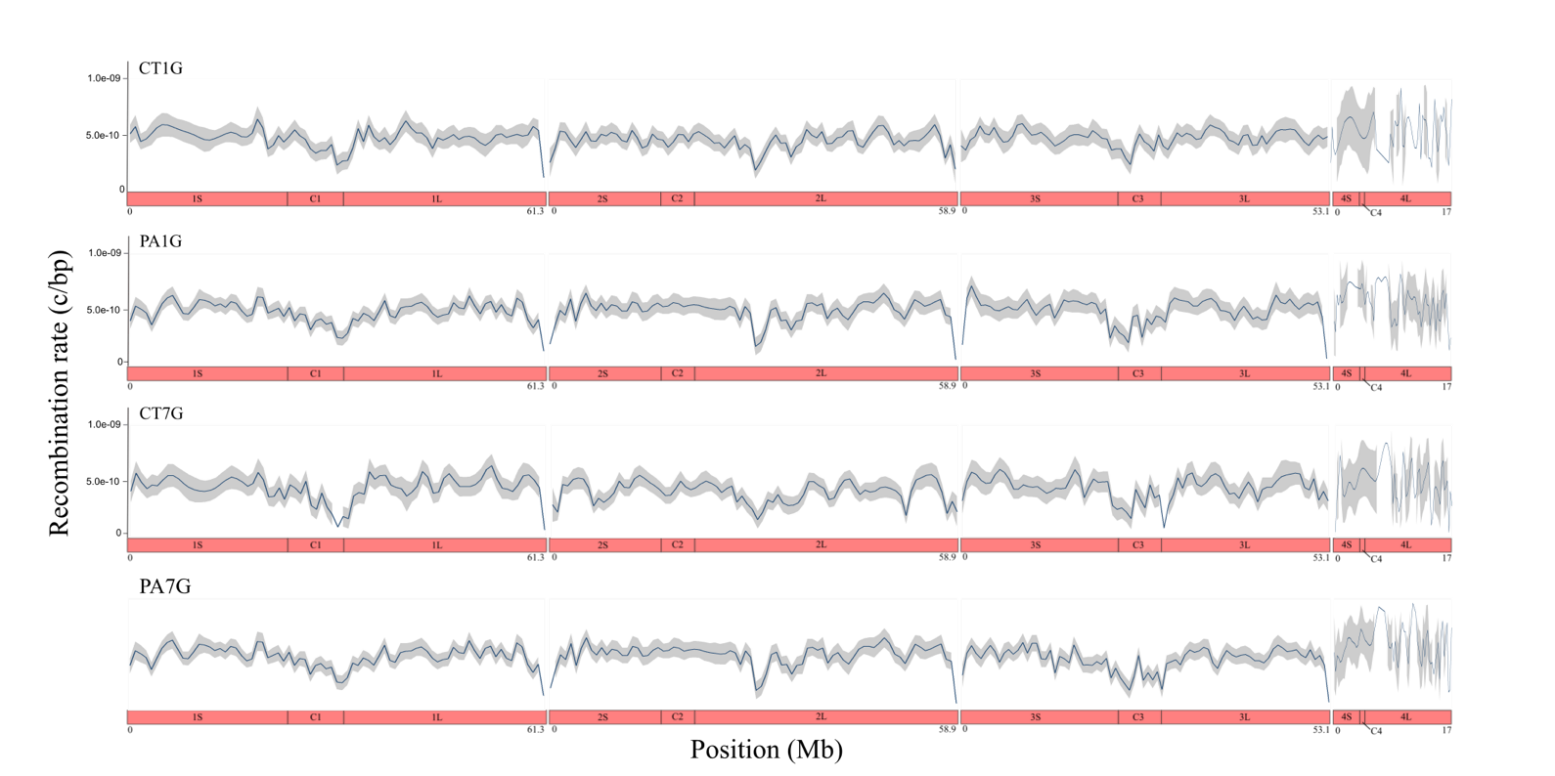


**Figure S2**. Genome-wide distribution of 10kb recombination rates comparison of Hessen ancestral pool (geography dataset) and experimental selection datasets: a) temperature, b) microplastics and c-e) Cadmium datasets.


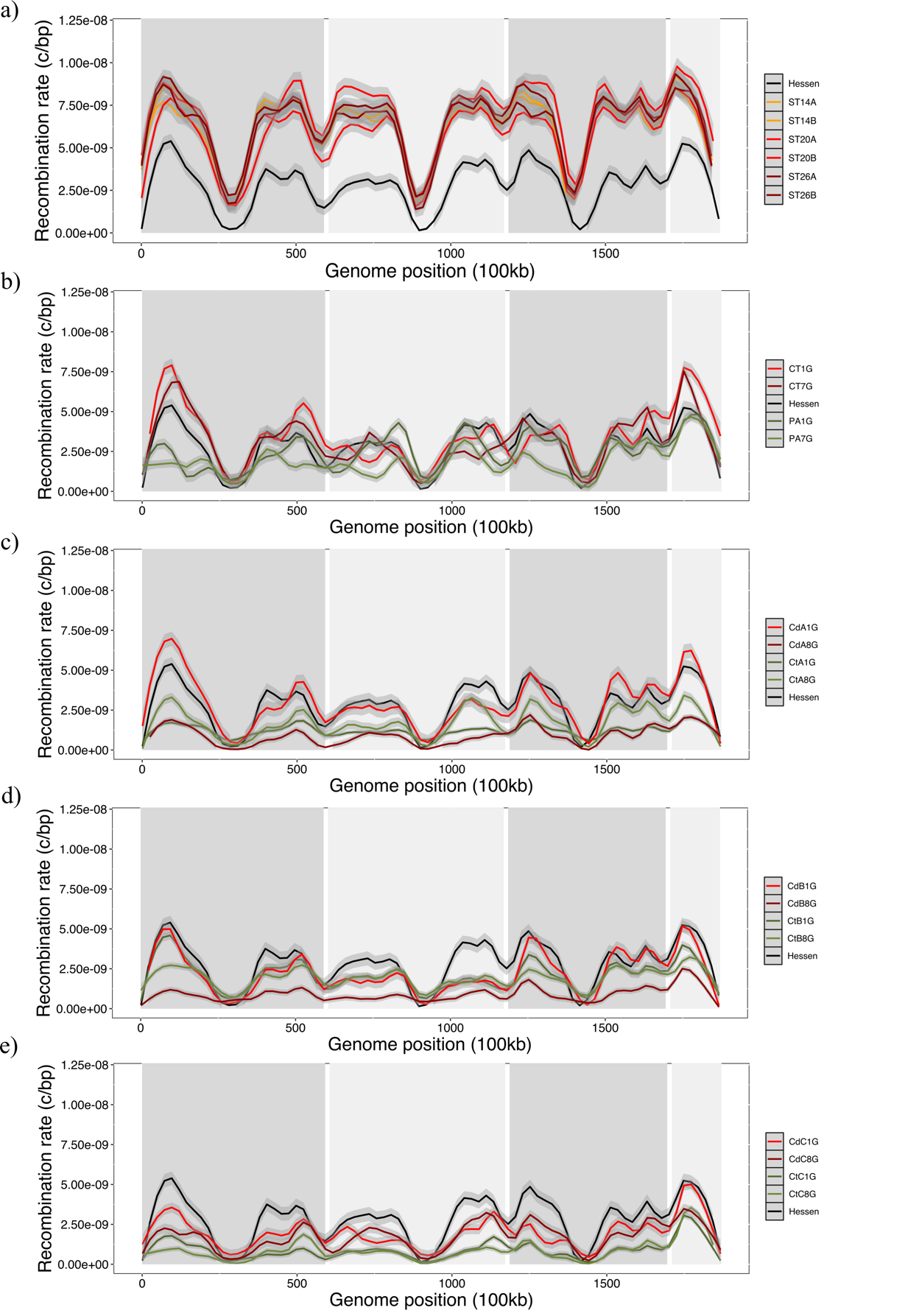


**Figure S3**. Plots showing the distribution of difference (Δ) in recombination rates between: a) 8^th^ and 1^st^ generation for control and Cadmium pools replicate C; and b) 7^th^ and 1^st^ generation for control and microplastics pools.

**
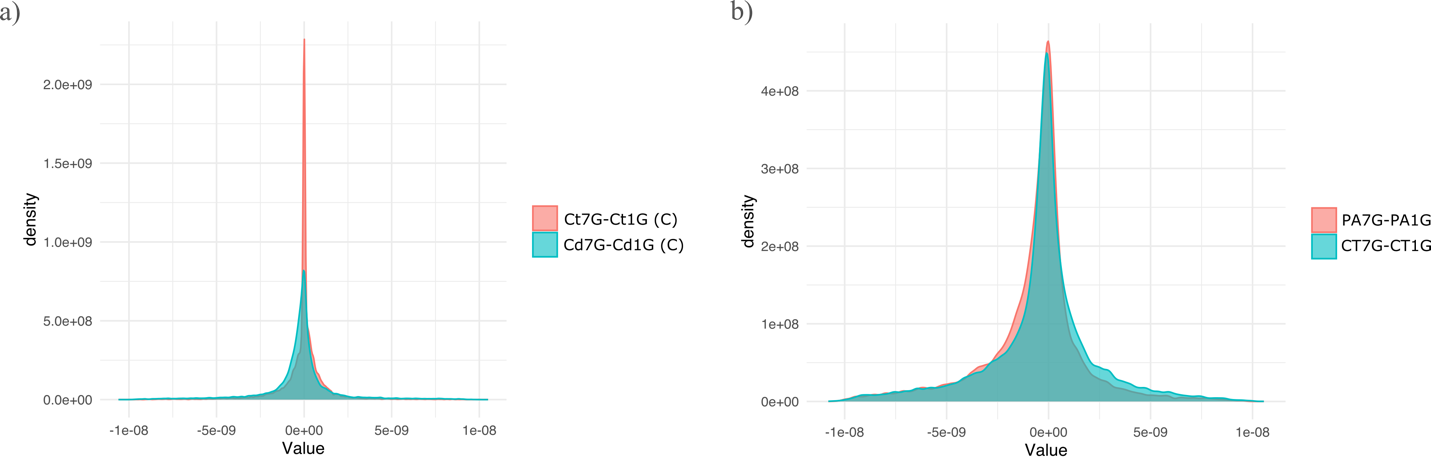
**

**Figure S4**. Correlation between high Δ differences of recombination rates (> 95% percentile) and their corresponding F_ST_ window estimate. a) Indicates correlations for Cadmium replicate A and B; and b) indicates correlations all temperature treatments for both replicates and ancestral pool from seasonality dataset (NP0).

a)

**
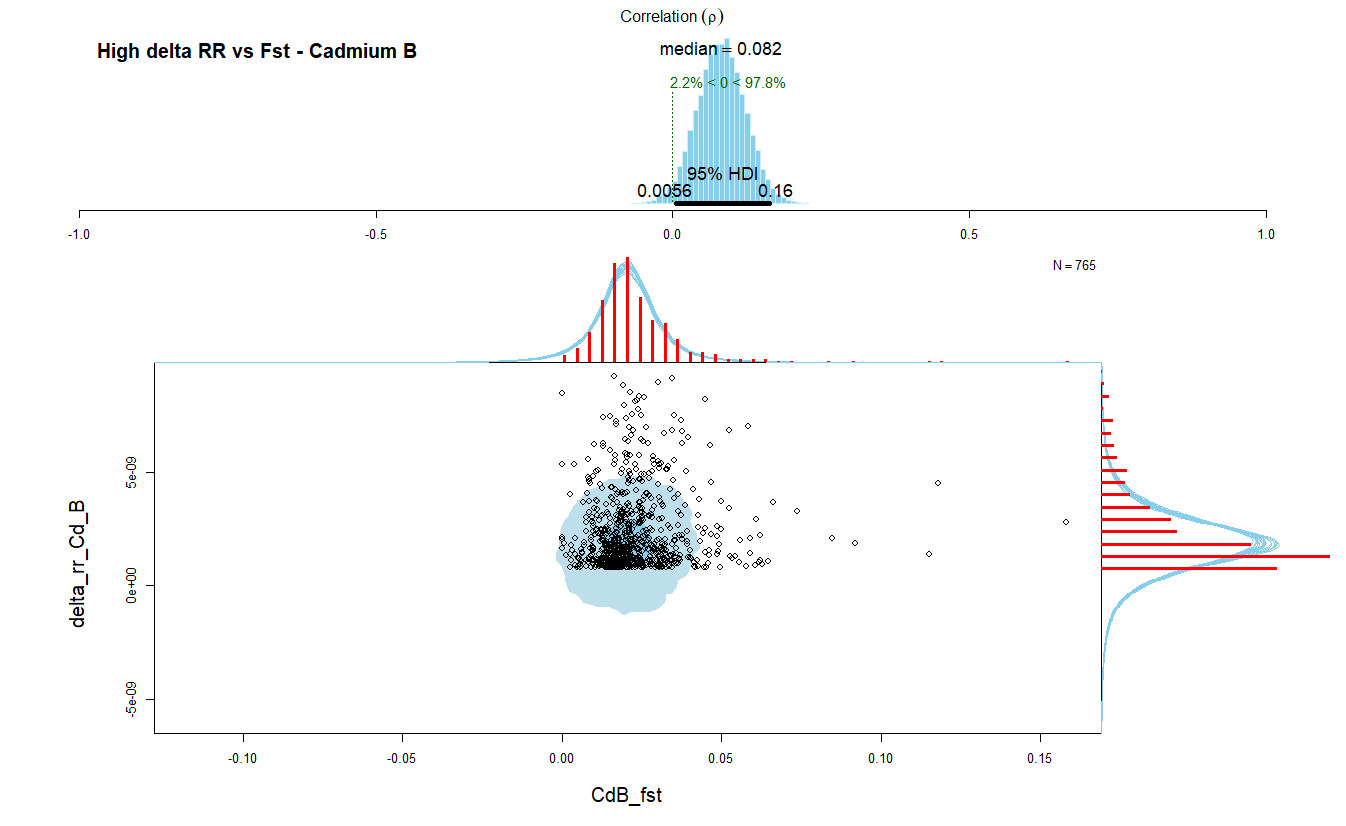

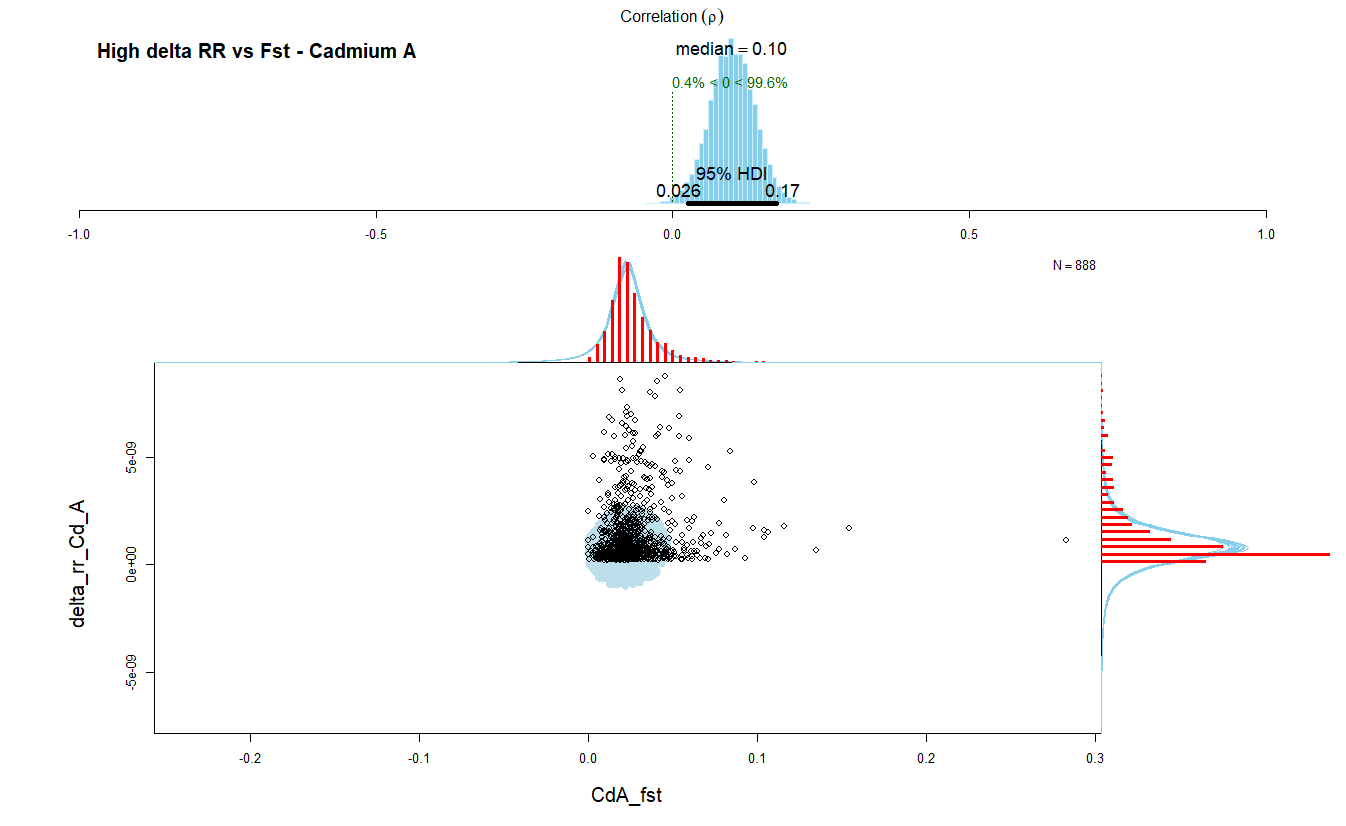
**

**\**

b)

**
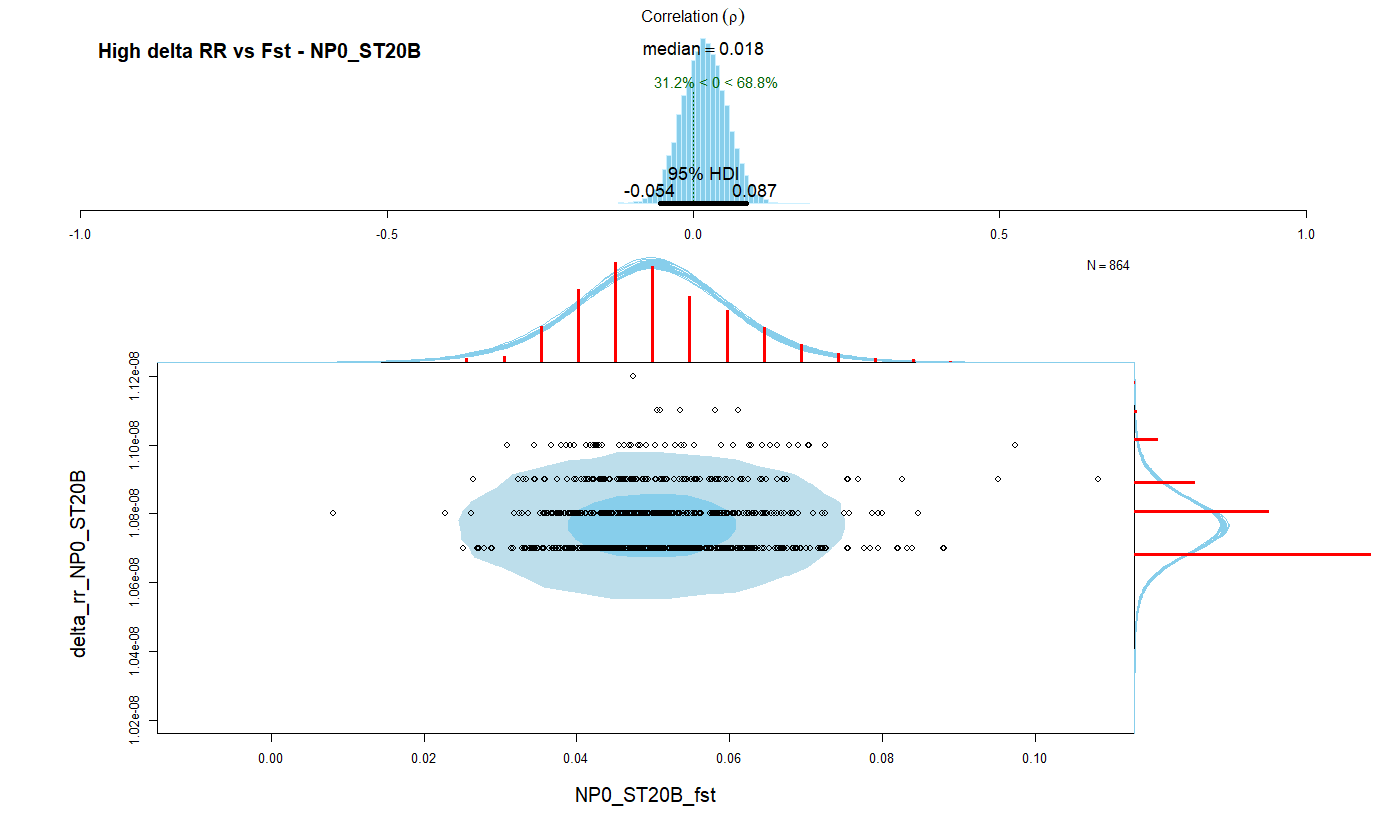

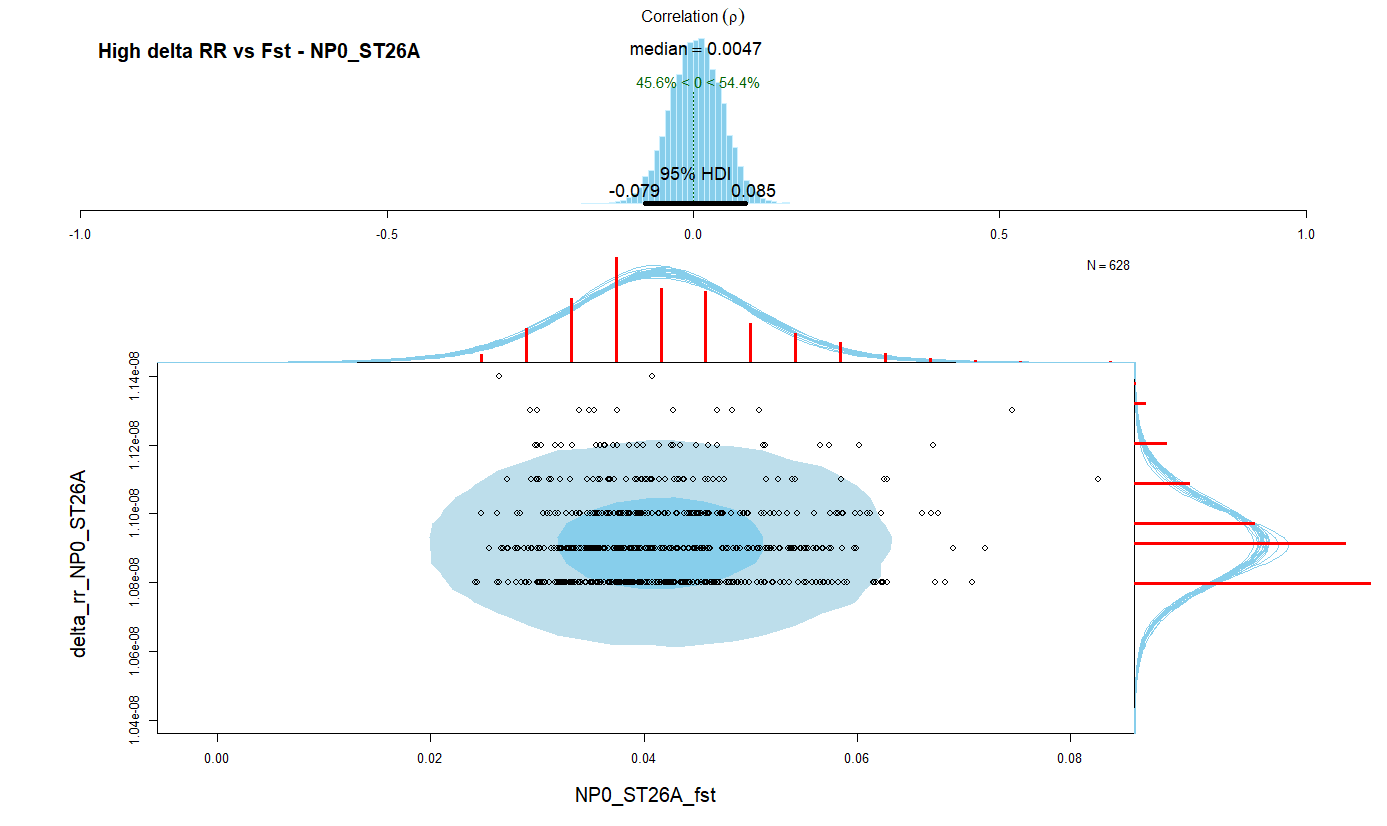
**
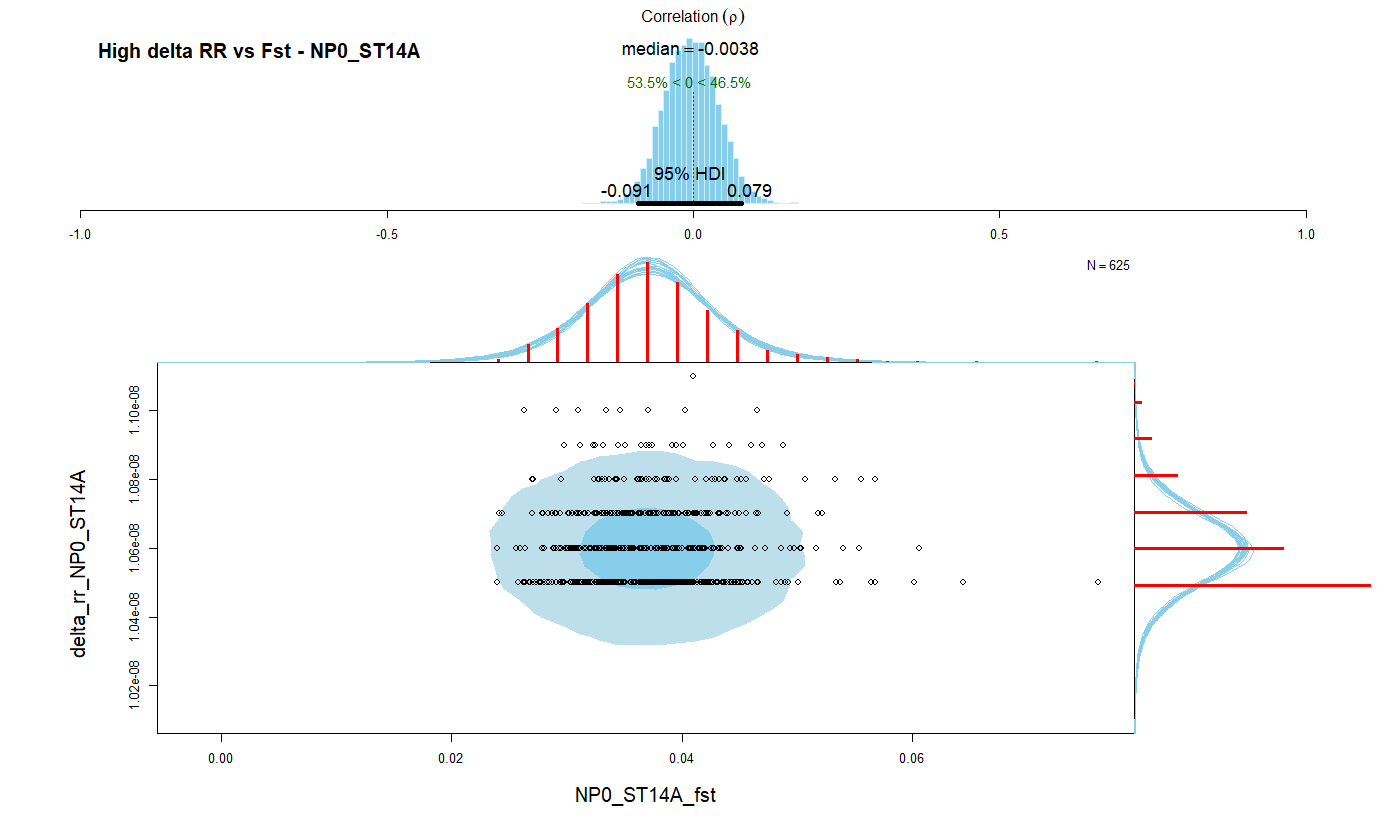

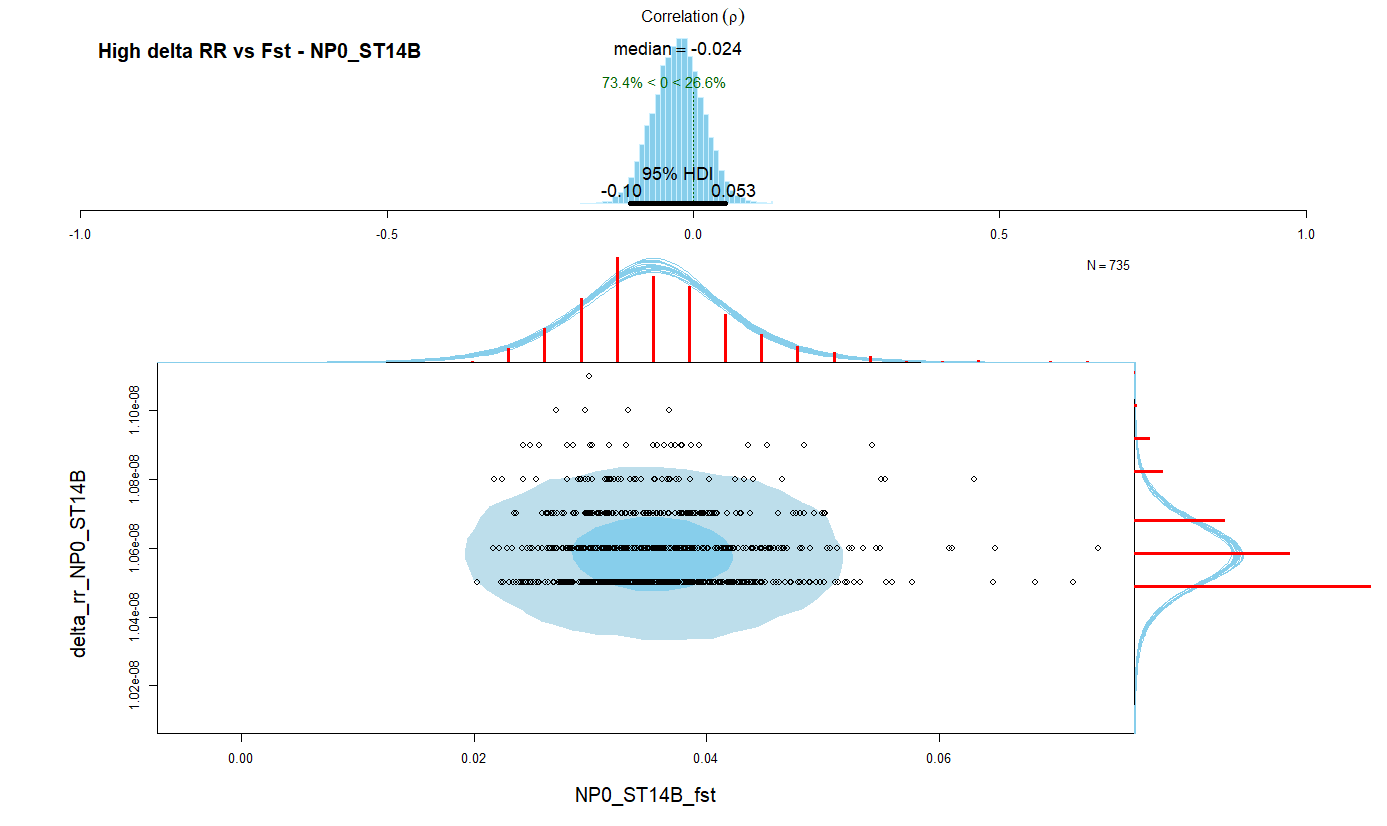
**
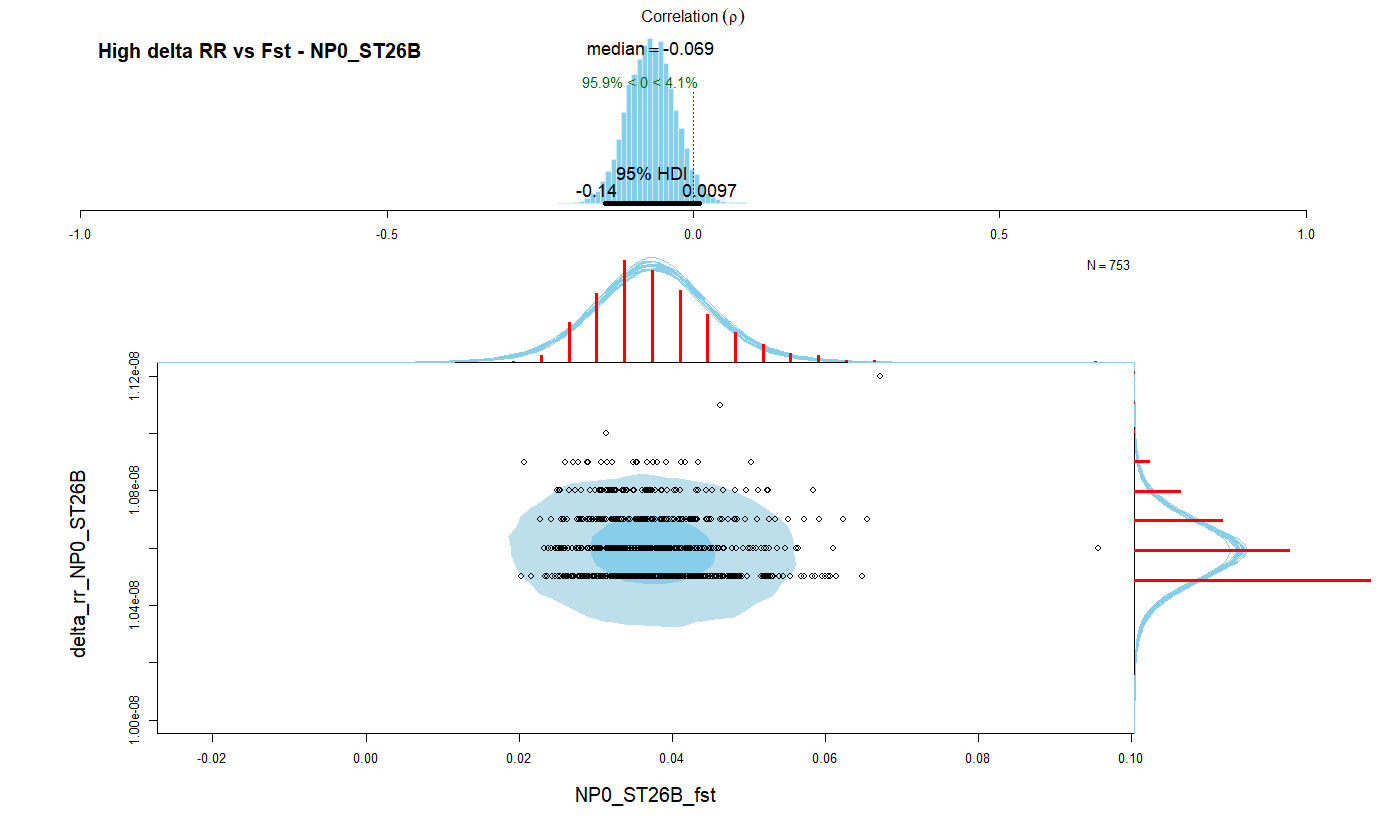
**

**
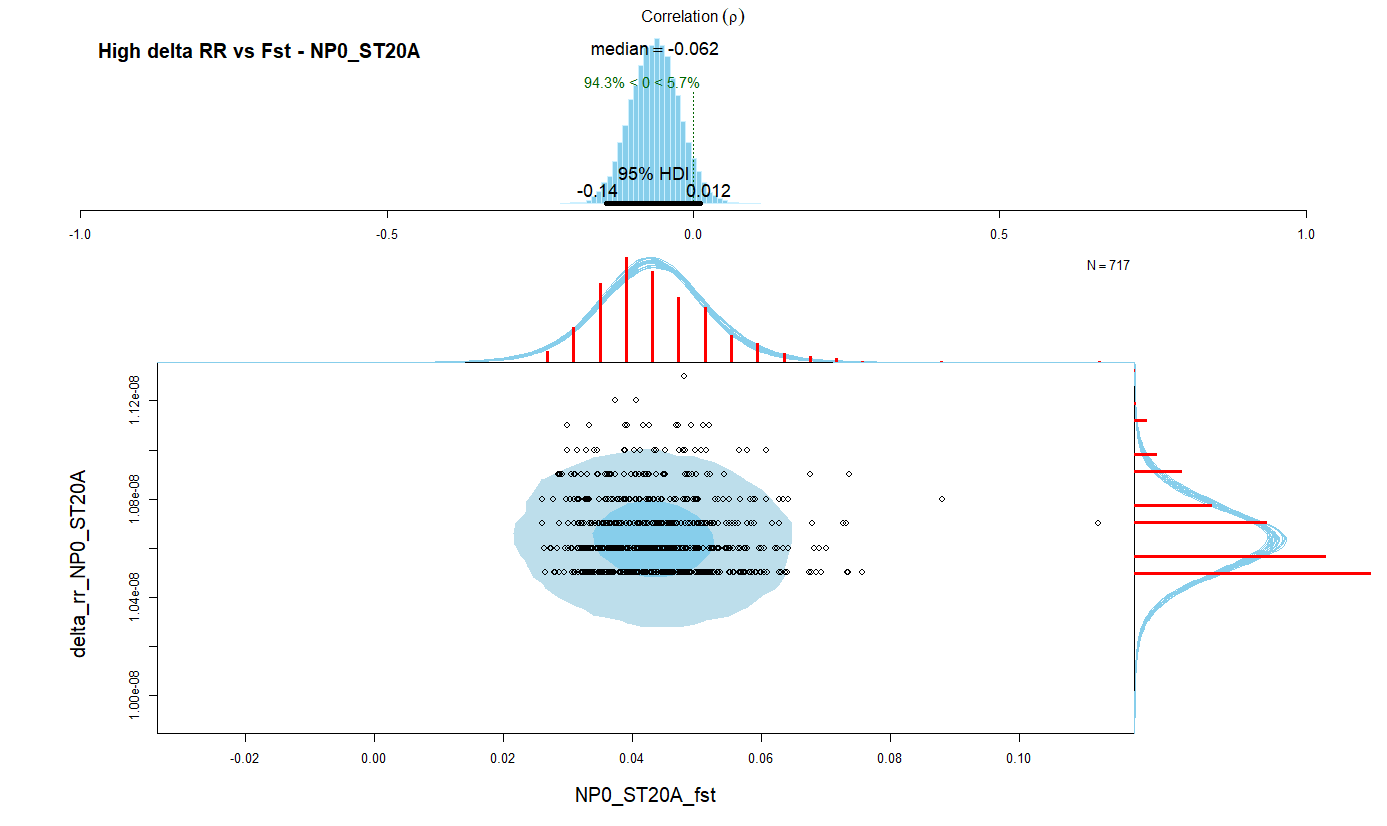
**

**Figure S5**. SEM models ﻿of hypothesised causal and covariance relationships for experimental datasets: a) temperature dataset; b) Cadmium dataset; and c) microplastics dataset. ﻿Fisher’s C indicates model fit, with *p*-values Degrees of freedom are marked with df; n = number of genomic windows included in the specific analysis; Values next to arrows show standardised estimates, with asterisk indicating the statistical significance of the relationship (*p < 0.05, **p < 0.01, ***p < 0.001). Black and red arrows indicate statistical significance causal and covariance relationships, respectively; Grey arrows indicates relationship that were not statistically significance.


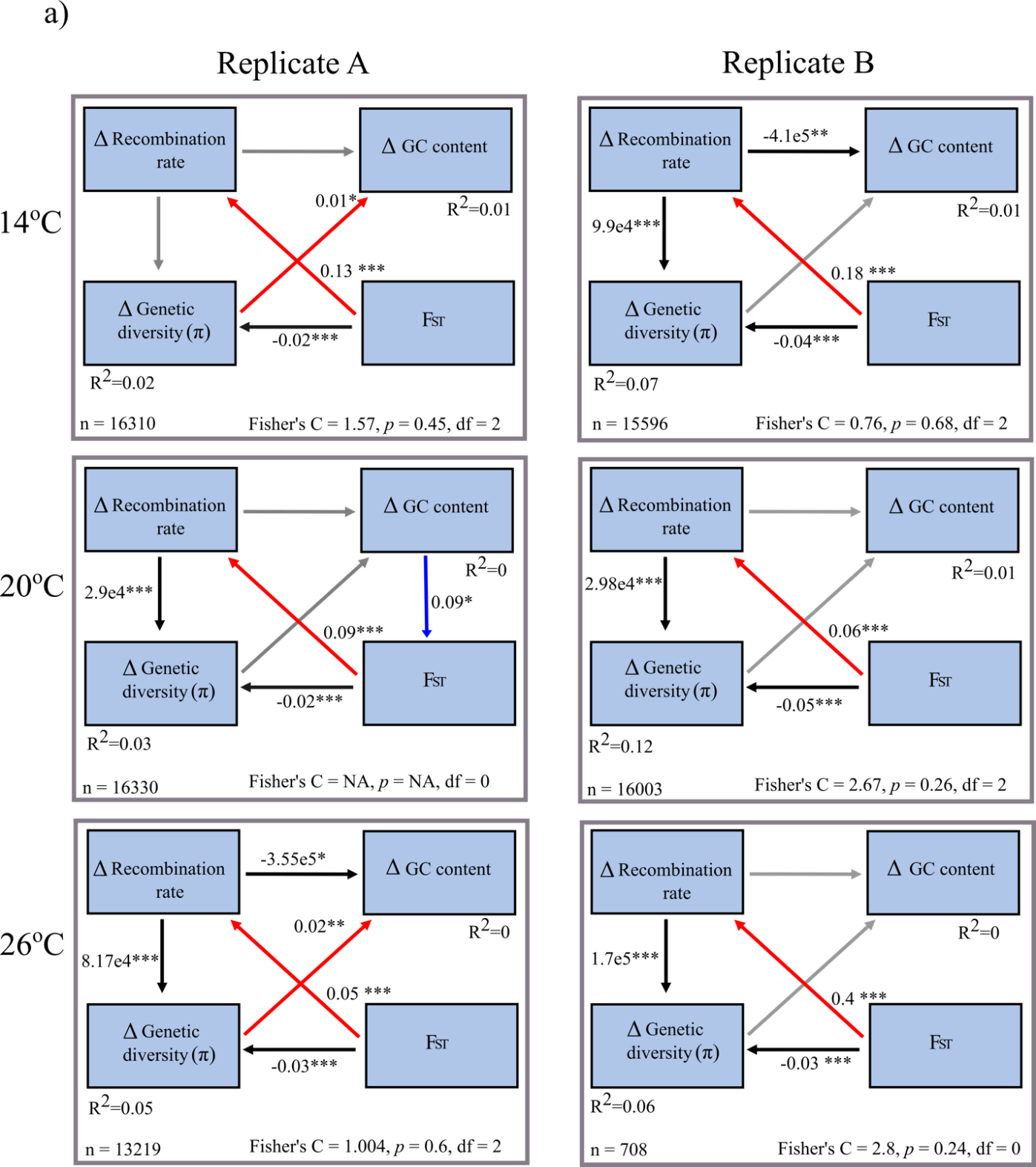


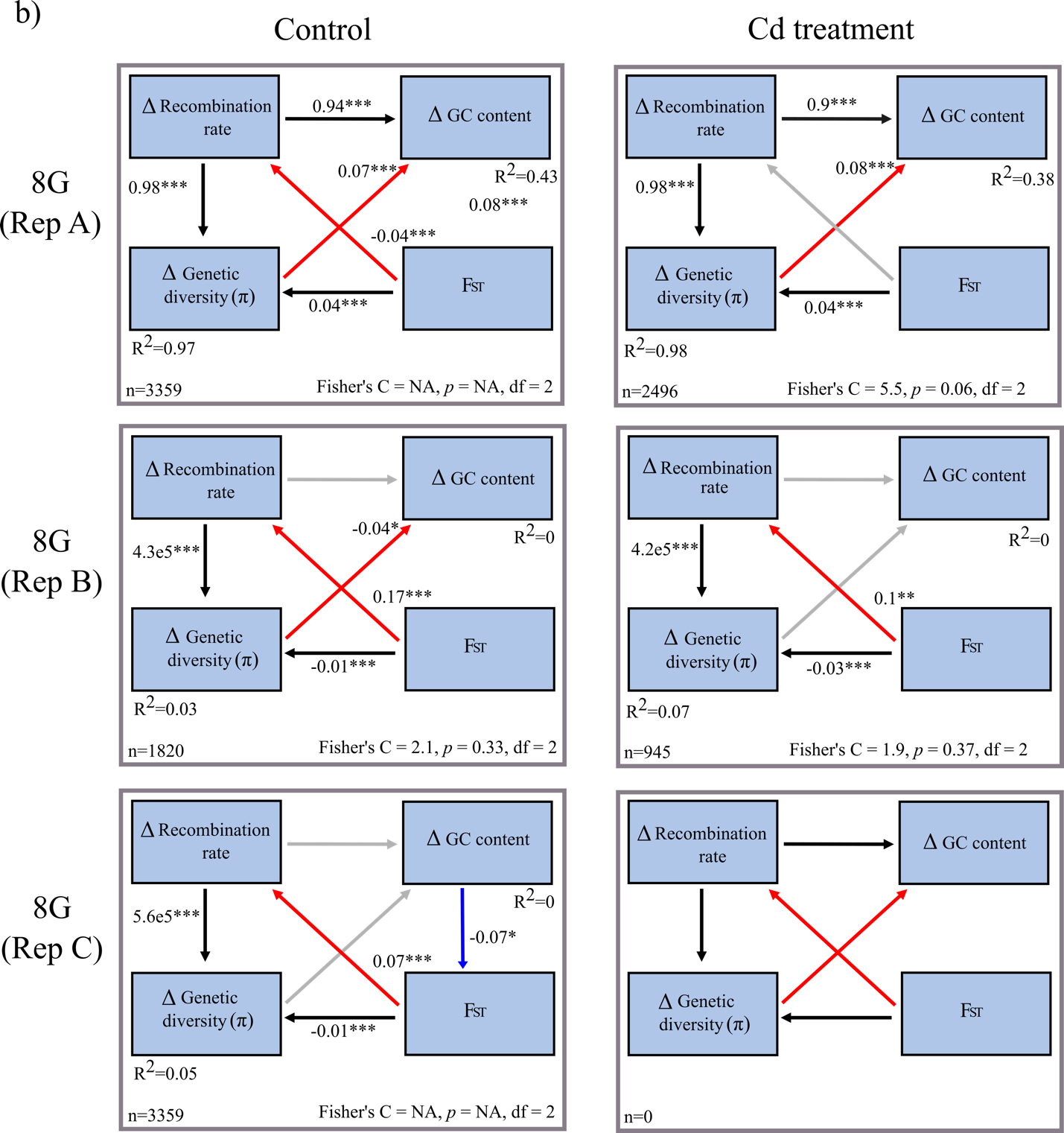

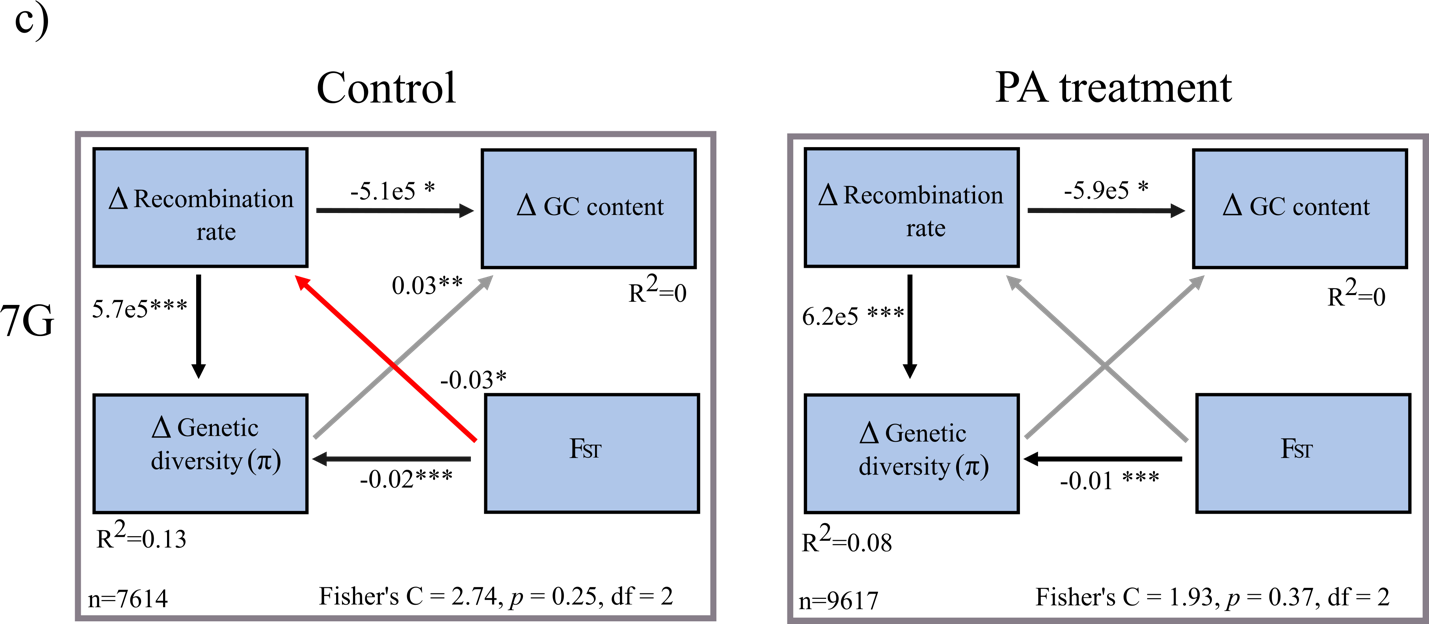


**Table S2**. SEM statistical support for causal and covariance relationships based on hypothetical model (Figure 1). Asterisks indicate the ﻿statistical significance of the relationship (*p < 0.05, **p < 0.01, ***p < 0.001). ~ indicates no statistical support. Analysis for Cadmium replicate C 8^th^ generation was not possible dur to missing data. RR = recombination rates, π = genetic diversity, GC = GC content.

|  | **Pool** | **Causal relationship** | | | **Covariance relationship** | | |
| --- | --- | --- | --- | --- | --- | --- | --- |
|  |  | RR- π | RR-GC | π - F_ST_ | RR- F_ST_ | π -GC | GC- F_ST_ |
| **Temperature** | 14^o^C Rep A | ~ | ~ | *** | *** | * |  |
|  | 20^o^C Rep A | *** | ~ | *** | *** | ~ |  |
|  | 26^o^C Rep A | *** | * | *** | *** | ** |  |
|  | 14^o^C Rep B | *** | ** | *** | *** | ~ |  |
|  | 20^o^C Rep B | *** | ~ | *** | *** | ~ |  |
|  | 26^o^C Rep B | *** | ~ | *** | *** | ~ |  |
| **Microplastics** |  |  |  |  |  |  |  |
|  | Control 7G | *** | * | *** | * | ~ |  |
|  | MP-PA 7G | *** | * | *** | ~ | ~ |  |
|  |  |  |  |  |  |  |  |
| **Cadmium** | Ct 8G Rep A | *** | *** | *** | ** | *** | *** |
|  | Cd 8G Rep A | *** | *** | *** | ~ | *** |  |
|  | Ct 8G Rep B | *** | ~ | *** | *** | * |  |
|  | Cd 8G Rep B | *** | ~ | *** | ** | ~ |  |
|  | Ct 8G Rep C | *** | ~ | *** | *** | ~ | * |
|  | Cd 8G Rep C | no data | no data | no data | no data | no data |  |
